# Melatonin Mediates Axillary Bud Outgrowth by Improving Nitrogen Assimilation and Transport in Rice

**DOI:** 10.3389/fpls.2022.900262

**Published:** 2022-07-13

**Authors:** Guo Yang, Xilin Wei, Zhongming Fang

**Affiliations:** ^1^Key Laboratory of Plant Resource Conservation and Germplasm Innovation in Mountainous Region (Ministry of Education), College of Agricultural Sciences, Guizhou University, Guiyang, China; ^2^Center of Applied Biotechnology, Wuhan University of Bioengineering, Wuhan, China

**Keywords:** melatonin, axillary bud, nitrogen, amino acid, *OsAAP14*, rice

## Abstract

Melatonin plays an important role in plant resistance to biotic and abiotic stresses. However, whether melatonin is involved in the regulation of plant architecture, such as the formation of axillary bud outgrowth or tillering, in rice remains unknown. Here, we found that different concentrations of melatonin influenced axillary bud outgrowth in rice, and moderate melatonin concentrations also alleviated the inhibition of axillary bud outgrowth in the presence of high concentrations of basic amino acids lysine and arginine. Furthermore, transcriptome analysis demonstrated that genes involved in nitrogen metabolism and phytohormone signal transduction pathways may affect axillary bud outgrowth, which is regulated by melatonin. We determined that the differentially expressed genes glutamine synthetase *OsGS2* and amino acid transporter *OsAAP14*, which are involved in nitrogen metabolism and are regulated by melatonin and basic amino acids, were the key regulators of axillary bud outgrowth in rice. In addition, we validated the functions of *OsGS2* and *OsAAP14* using rice transgenic plants with altered axillary bud outgrowth and tillers. Taken together, these results suggest that melatonin mediates axillary bud outgrowth by improving nitrogen assimilation and transport in rice.

## Introduction

Melatonin (N-acetyl-5-methoxytryptamine) is an indolic compound first reported in bovine pineal glands (Lerner et al., 1958). Recently, melatonin has been widely used as a plant biostimulator to regulate growth and enhance plant resistance to both biotic and abiotic stresses. On the one hand, melatonin is involved in multiple plant biological processes, such as leaf senescence (Zhang et al., 2017), photosynthesis (Tan et al., 2019), flowering (Arnao and Hernández-Ruiz, 2020), fruit ripening (Onik et al., 2021), root induction (Yang et al., 2021), and other growth and developmental processes. On the other hand, melatonin confers tolerance to plants against several abiotic stresses, including cold (Bajwa et al., 2014), high temperatures (Larkindale and Huang, 2005), heavy metals (Posmyk et al., 2008), salinity (Vafadar et al., 2020), oxidative stress (Bahcesular et al., 2020), and drought (Wang et al., 2021), and various biotic stressors, including bacteria (Lee et al., 2014), fungi (Mandal et al., 2018), and viruses (Sofy et al., 2021). Nonetheless, whether melatonin is involved in the regulation of plant architecture, such as the formation of axillary bud outgrowth or tillering, in rice still remains unclear.

Tillering is an important agronomic trait that contributes to grain yield, and also a model system for the study of branching in monocotyledonous plants. Tillering occurs in a two-stage process: the formation of an axillary bud at each leaf axil and its subsequent outgrowth (Li et al., 2003). Tillering is regulated by several phytohormones and external environmental factors. The phytohormone auxin affects the formation of tillering buds, and the auxin transporter genes *OsPIN1* (Xu et al., 2005), *OsPIN2* (Chen et al., 2012), *OsPIN5b* (Lu et al., 2015), and *OsPIN9* (Hou et al., 2021b) regulate rice tillering by altering the local distribution of auxin. Strigolactones (SLs) regulate rice tillering by inhibiting the elongation of rice axillary buds (Umehara et al., 2008). The synthetic genes *D27* (Lin et al., 2009), *D17* (Zou et al., 2005), *D10* (Arite et al., 2007), receptor gene *D14* (Arite et al., 2009), and downstream signaling genes *D53* and *FC1* (Jiang et al., 2013; Zhou et al., 2013) in the SL pathway are involved in the regulation of rice tillering. Recent evidence has revealed abscisic acid (ABA)-repression of lateral bud outgrowth in *Arabidopsis* (Yao and Finlayson, 2015). A high concentration of ABA acts as a growth inhibitor to induce axillary bud dormancy under adverse environmental conditions (Gonzalez-Grandio et al., 2017). In the ABA signaling pathway, studies have shown that OsNCED1 plays important roles in ABA biosynthesis and inhibition of bud outgrowth in rice (Luo et al., 2019). In addition, ABA can also interact with other phytohormones to affect plant growth and development. SLs promote ABA biosynthesis in shoot basal part, whereas ABA inhibits SL biosynthesis, revealing SL and ABA biosynthesis are closely integrated to coordinately repress tillering in rice (Liu et al., 2020). Besides, ABA and GA antagonistically regulate the growth of rice root and the increase of tillering (Lin et al., 2020). However, cytokinins (CKs) promote the elongation of axillary buds (Duan et al., 2019), but the rice CK oxidase genes *OsCKX2* (Yeh et al., 2015), *OsCKX4* (Gao et al., 2014), and *OsCKX9* (Duan et al., 2019) negatively regulate tillering. Recently, brassinosteroids (BRs) have also been shown to play significant roles in the regulation of tillering, with OsBZR1–DLT–RLA1 signaling complex required for axillary bud outgrowth (Fang et al., 2020).

Nitrogen is an important external environmental factor required for axillary bud outgrowth, which influences tillering and grain yield in rice. Nitrate, ammonium, and several amino acids can be utilized by plants after being converted into other organic nitrogen forms by various processes. Reports suggest that elevated levels of nitrate or ammonium ions in the soil can promote rice tillering (Sasaki et al., 2002). Recently, it is reported that different nitrogen concentrations significantly influence the outgrowth of axillary buds for rice tillering (Wang et al., 2020). The lengths of axillary buds were shorter under lower nitrogen concentrations 0.5 and 1.0 mm than those under the optimal nitrogen concentration (2.0 mm), whereas the lengths of axillary buds were longer under 5.0 and 10.0 mm nitrogen concentrations. Remarkably, the axillary buds were shorter when the nitrogen concentration reached 15.0 mm (Wang et al., 2020). Nitrogen displays a significant promoting effect on tiller development by regulating nitrogen-metabolism and endogenous hormone levels (Liu et al., 2011). Farmers usually apply nitrogen fertilizers to enhance the number of tillers, and a sufficient application of N could increase rice tillers and panicles (Zhang et al., 2013). Therefore, increasing nitrogen application rate in rice with high tillering capacity could compensate for the yield reduction of low planting density (Huang et al., 2013).

Furthermore, NITRATE TRANSPORTER 1/PEPTIDE TRANSPORTER family (NPF) genes *OsNPF7.1*, *OsNPF7.2*, *OsNPF7.3*, *OsNPF7.7*, and *OsNPF8.20* positively regulate rice tiller number, whereas *OsNPF7.4* negatively regulates rice tillering (Fang et al., 2013, 2017; Huang et al., 2018, 2019; Wang et al., 2018). Importantly, *OsNPF6.5* (*OsNRT1.1B*) is a key gene controlling nitrogen uptake and utilization, and influence grain yield by regulating rice tillering (Hu et al., 2015). Recently, it was reported that *OsNPF5.16*, a nitrate transporter gene with natural variation in its promoter sequence, is essential for rice tillering and yield (Wang et al., 2022b). In addition, the glutamine synthetase is a key point in nitrogen assimilation where ammonium is incorporated into glutamine, providing the precursor for production of all amino acids, nucleic acids, and chlorophylls (Kissen et al., 2010). In rice, there are three genes that encode cytosolic GS1 (*OsGS1;1*, *OsGS1;2* and *OsGS1;3*), and one gene encodes chloroplastic GS2 (*OsGS2*; James et al., 2018). Mutants of cytosolic glutamine synthetase 1;2 reduce yield by reducing tillering and panicle number (Funayama et al., 2013). Omics analysis was then performed in the basal part of the seedlings and showed that the reduction in the number of tillering in *OsGS1;2* mutants is due to a change in metabolic balance and is independent of the regulation of tillering by strigolactones (Ohashi et al., 2015). Besides, deletion of *OsGS1;2* reduces *IPT4* gene expression at the base, which is shown to reduce the amount of tZ-type cytokinins, resulting in reduced tiller number (Ohashi et al., 2017), and deletion of *OsGS1;2* also reduces expression of the *fructose-1,6-bisphosphatase* gene (Ohashi et al., 2018). Furthermore, it has been shown that *OsGS2* regulate axillary bud growth and tiller number in rice (Wang et al., 2020).

Amino acid transporters also play important roles in rice tillering. OsAAP1 primarily mediates neutral amino acids, such as proline, alanine, and tyrosine, and positively regulates rice tillering (Ji et al., 2020). OsAAP3 and OsAAP5 predominantly transport lysine (Lys) and arginine (Arg), and negatively regulate rice tillering (Lu et al., 2018; Wang et al., 2019). OsAAP4 regulates the transport of neutral amino acids valine and proline, and plants over-expressing *OsAAP4* exhibit increased axillary bud elongation under different neutral amino acid concentrations (Fang et al., 2021). OsAAP6 facilitates the uptake and transport of threonine, serine, glycine, alanine, proline, and acidic amino acids by rice roots (Peng et al., 2014). In rice, low concentrations of Lys and Arg promote the elongation of axillary buds, whereas slightly higher concentrations inhibit axillary bud elongation (Lu et al., 2018). However, the molecular mechanism underlying the inhibition of rice axillary bud elongation by high concentrations of Lys and Arg remains to be elucidated. Therefore, this study aimed to determine the effects of different concentrations of melatonin, Lys, and Arg on the growth of axillary buds in rice. We further identify the genes involved in the regulation of rice axillary bud elongation and validate their functions using transgenic plants.

## Materials and Methods

### Plant Material and Growth Conditions

The germinated seeds of wild-type (WT) rice cultivar ZH11 (*Oryza sativa* L. ssp. *japonica*) were grown in basic rice nutrient solution for 14 days. The basic nutrient solution was composed of 1.0 mm NH_4_NO_3_, 0.32 mm NaH_2_PO_4_, 0.51 mm K_2_SO_4_, 1.0 mm CaCl_2_, 1.65 mm MgSO_4_, 8.9 μm MnSO_4_, 0.5 μm Na_2_MoO_4_, 18.4 μm H_3_BO_3_, 0.14 μm ZnSO_4_, 0.16 μm CuSO_4_, and 40.0 μm FeSO_4_. The seedlings were then transferred in a greenhouse and treated with basic nutrient solutions supplemented with different concentrations of melatonin (0 μm, 0.1 μm, 1 μm, 10 μm, 100 μm, and 1,000 μm), 1.5 mm Lys + 0.5 mm Arg, or 1.5 mm Lys + 0.5 mm Arg + 1 μm melatonin for 21 days. The solutions were renewed every 3 days, and the greenhouse maintained a 16 h-light (32°C)/8 h-dark (25°C) photoperiod. At 35 days after germination, the length of first and second axillary buds of ZH11 plants were measured. Simultaneously, axillary buds and basal parts from each treatment were excised and immediately frozen in liquid N_2_ for total RNA extraction. Thereafter, total RNA was extracted using TRIzol reagent (Vazyme, Nanjing, China) for RNA-Seq. The raw data collected from RNA-seq are available at the National Center for Biotechnology Information (NCBI): https://dataview.ncbi.nlm.nih.gov/object/PRJNA820302?reviewer=rlhbj4ujtu845m90qfffvupk0p.

### Library Preparation for RNA-Seq and Data Processing

A total of 1 μg RNA per sample was used for constructing cDNA libraries. cDNA library preparation and sequencing were carried out by Beijing Nuohe Zhiyuan Technology Co. Ltd. (Nuohe, Beijing, China) following manufacturer’s recommendations. The clustering of the index-coded samples was performed on a cBot Cluster Generation System using TruSeq PE Cluster Kit v3-cBot-HS (Illumina, San Diego, United States) according to the manufacturer’s instructions. After cluster generation, the libraries were sequenced on an Illumina Novaseq platform and 150-bp paired-end reads were generated.

Raw reads in the FASTQ format were first processed using in-house Perl scripts, and then processed reads were obtained by removing reads containing adapter and poly-N and low-quality reads from the raw data. Subsequently, the clean reads were aligned to the Nipponbare reference genome (Ensemble_37) using Hisat2 v2.0.5. To determine the expression levels of genes in the samples, fragments per kilobase of transcript per million reads (FPKM) were calculated using FeatureCounts v1.5.0-p3. Then, principal component analysis was performed online,^1^ and expression correlations between samples were calculated using the cor function in R software.

### Gene Ontology and KEGG Enrichment Analysis of Differentially Expressed Genes

Gene Ontology (GO) enrichment analysis of differentially expressed genes (DEGs) was performed using the cluster Profiler R package, wherein gene length bias was corrected. GO terms with corrected value of *p* < 0.05 were considered significantly enriched. We used the clusterProfiler R package to test the statistical enrichment of DEGs in the Kyoto Encyclopedia of Genes and Genomes (KEGG) pathways.

### Quantitative Real-Time Polymerase Chain Reaction

Total RNA was extracted using Trizol reagent according to the manufacturer’s instructions (Vazyme, Nanjing, China). First-strand cDNA were synthesized using 3 μg of total RNA extracted from each sample using MLV reverse transcriptase (Vazyme, Nanjing, China). Quantitative real-time polymerase chain reaction (qRT-PCR) was performed in a 20 μl reaction volume containing 10 μl 2 × SYBR Green Mix (Vazyme, Nanjing, China), 1 μl cDNA solution, and 1 μl gene-specific primers (10 μm) under the following conditions: 94°C for 2 min (1 cycle), 94°C for 30 s, 55°C for 30 s, and 72°C for 30 s (40 cycles), and 72°C for 1 min (1 cycle) using 7,500 RT qPCR system (Applied Biosystems, Foster City, United States). The primers used for qPCR are listed in Supplementary Table 1.

### Vector Construction and Transgenic Plant Generation

To construct *OsGS2* and *OsAAP14* promoter-GUS vectors, 2,238-bp and 2,300-bp fragments upstream of the start codon (ATG) of *OsGS2* and *OsAAP14*, respectively, were inserted into the pCAMBIA1391Z vector using *Hin*dIII and *Nco*I restriction enzymes. To construct *OsGS2*- and *OsAAP14*-overexpression (OE) vectors, 1,287-bp *OsGS2* (LOC_Os04g56400) and 1,410-bp *OsAAP14* (LOC_Os04g56470) cDNA sequences were inserted downstream of the CaMV 35S promoter in the pCAMBIA1306 vector using *Kpn*I and *Xba*I restriction enzymes. To generate the *OsGS2*-RNAi vector, two 217-bp fragments of *OsGS2* cDNA were inserted downstream of the Ubi-1 promoter in the rice RNAi vector pTCK303 using *Bam*HI/*Kpn*I and *Spe*I/*Sac*I, respectively. The *OsAAP14* CRISPR plasmid was constructed using the CRISPR/Cas9-based multiplex genome editing system for monocot and dicot plants (Ma et al., 2015). These vectors were then transformed into *Agrobacterium tumefaciens* strain *EHA105* and introduced into the calli of ZH11, following which T0 plants were selected using 50 mg l^−1^ hygromycin. Homologous T2 lines were used for all experiments.

### Gus Signal Analysis of the *OsAAP14* and *OsGS2* Promoter

To analyze *OsGS2* and *OsAAP14* expression levels in the presence of different melatonin concentrations, three-week-old *OsGS2* and *OsAAP14* promoter-GUS transgenic plants were treated with basic solutions supplemented with different concentrations of melatonin (0 μm, 0.1 μm, 1 μm, and 10 μm), 1.5 mm Lys + 0.5 mm Arg, or 1.5 mm Lys + 0.5 mm Arg + 1 μm melatonin for 2 h and 24 h. GUS staining was performed according to a previously described histochemical staining method (Fang et al., 2017). The stained samples were then observed using a stereomicroscope (OLYMPUS SZX16, Tokyo, Japan). To make sections, the stained tissues were rinsed and fixed in FAA at 4°C for 24 h, gradually dehydrated with ethanol for 15 min each time, and washed twice with 100% ethanol for 30 min each time. Finally, the samples were embedded in Spurr resin, and ultramicrotome sections (2–8 μm) were applied onto poly-l-lysine-coated slides with glass knives. The sections were observed using Zeiss Axio Imager M2 (Carl Zeiss AG, Oberkochen, Germany).

### FITC-Labeled Amino Acid Uptake Assay

Amino acids with FITC (Arg-FITC, Lys-FITC) were synthesized by Yuan Peptide Biotechnology Company, Nanjing, China. Then rice etiolated seedlings were prepared from ZH11, *OsGS2,* and *OsAAP14* transgenic plants. Fluorescence was detected after culturing seedlings of *OsAAP14* and *OsGS2* transgenic plants with FITC-labeled amino acids Arg for 2 h and 6 h, and Lys for 2 h and 6 h. Finally, fluorescence was observed using a fluorescence analyzer (Qinxiang, Shanghai, China).

### Determination of Free Amino Acids and Chlorophyll Content in Rice

To analyze the effects of *OsGS2* and *OsAAP14* expression levels on nitrogen metabolism and photosynthetic pathway, five-week-old *OsGS2* and *OsAAP14* transgenic plants were taken for total free amino acids and total chlorophyll content. Total free amino acid content was measured by the ninhydrin method (Fang et al., 2013). Total chlorophyll content was determined according to Luo et al. (2022).

### Statistical Analyses

The statistical chart were performed using GraphPad Prism 7, and the heatmaps were performed using TBtools. For multiple comparisons, Duncan’s multiple range test was performed using SPSS software, indicating significant difference at *p* < 0.05.

## Results

### Exogenous Low Concentration Melatonin Promoting Axillary Bud Elongation

**Figure 1:**
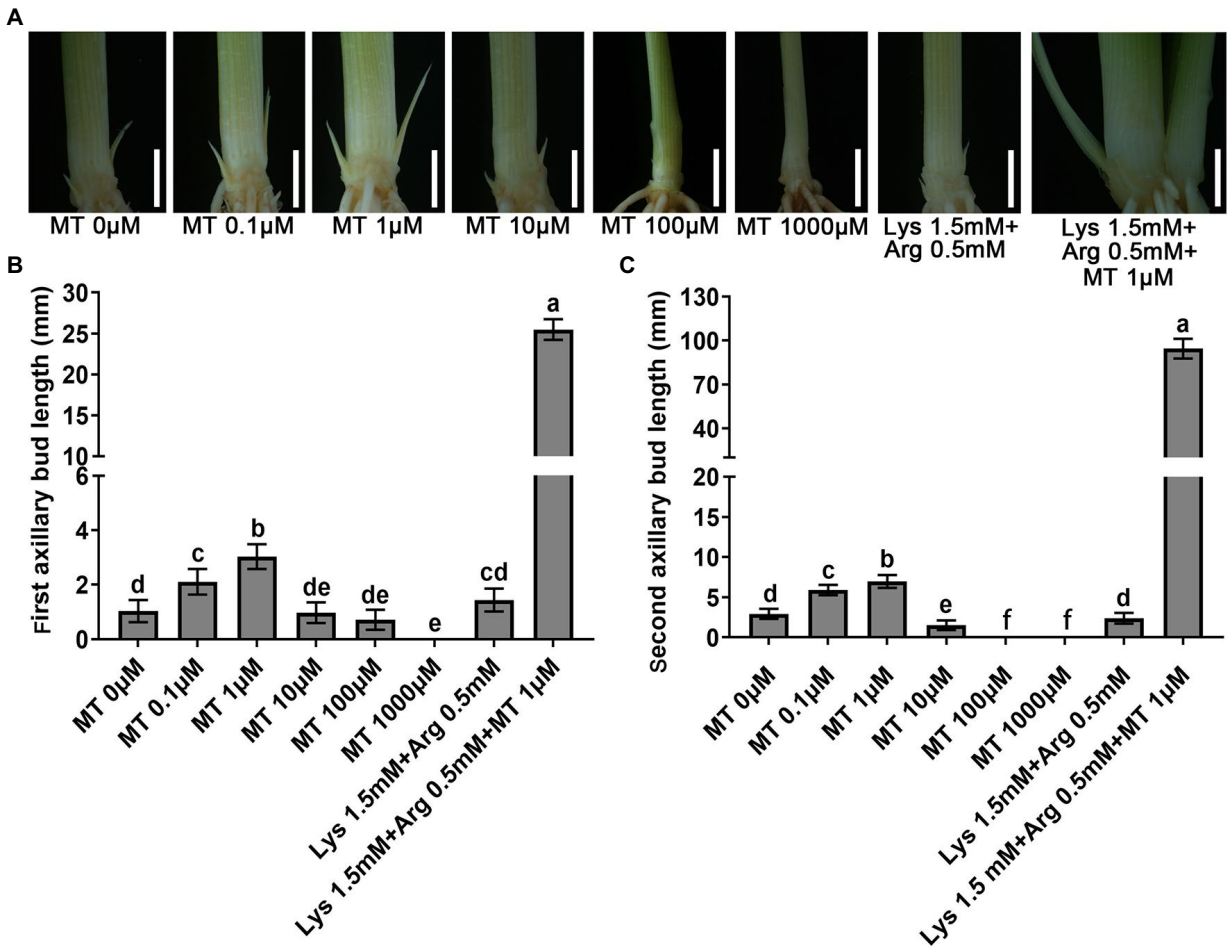
Phenotypes of outgrowth axillary bud under different concentrations of melatonin and amino acids treatments. **(A)** Phenotypes of outgrowth axillary buds under basic nutrient solutions supplemented with different concentrations of melatonin (0 μm, 0.1 μm, 1 μm, 10 μm, 100 μm, and 1,000 μm), 1.5 mm Lys + 0.5 mm Arg, or 1.5 mM Lys + 0.5 mM Arg + 1 μm melatonin. Statistical analysis of the first **(B)** and second **(C)** axillary bud length in **(A)**. MT represents melatonin, Lys represents lysine, and Arg represents arginine. Values are means ± SD (*n* > 20), and the significance levels of different lowercase letters were as follows, *p* < 0.05. Scale bar = 3 mm.

To investigate the effects of melatonin on tillering in rice, two-week-old ZH11 seedlings were cultured in basic nutrient solution supplemented with different concentrations of melatonin for 21 days, and the length of first and second axillary buds was measured. The results showed that low concentrations of melatonin (0–1 μm) promoted the elongation of the first and second axillary buds in rice, but higher concentrations (10–1,000 μm) inhibited bud elongation (Figures 1A–C). Furthermore, 1.5 mm Lys + 0.5 mm Arg inhibited the elongation of both first and second axillary buds (Figure 1). However, when these two amino acids were added with 1 μm melatonin, the inhibitory effect of the amino acids on rice axillary buds was not only alleviated but also the elongation of axillary buds increased. The effect of 1.5 mm Lys, 0.5 mm Arg and 1 μm melatonin on axillary bud elongation was stronger than that of 1 μm melatonin alone (Figure 1). These results demonstrated that melatonin plays an important role in axillary bud outgrowth in rice, and can alleviate the inhibition caused by high concentrations of basic amino acids on axillary bud outgrowth.

### Transcriptome Profiles of Axillary Buds and Basal Parts of Plants Grown Under Different Melatonin and Amino Acid Concentrations

To investigate the regulatory mechanisms underlying rice axillary bud outgrowth by melatonin, we sampled a mixture of the first and second axillary buds and basal parts of 35-day-old rice seedlings grown under different concentrations of melatonin for RNA-Seq analysis. We identified 4,480 DEGs in the axillary buds of plants treated with 0.1 μm melatonin compared with those treated with 0 μm melatonin, and 4,612 DEGs in the axillary buds of plants treated with 1 μm melatonin compared with those treated with 0.1 μm melatonin (Supplementary Figure S1A). Furthermore, 7,047 and 1,519 DEGs were detected in 10 μm melatonin-treated axillary buds compared with 1 μm and 0.1 μm melatonin-treated axillary buds, respectively (Supplementary Figure S1A). In addition, 2086 upregulated and 2,526 downregulated DEGs were detected in the axillary buds of plants treated with 0.1 μm melatonin compared with those treated with 1 μm melatonin (Supplementary Figure S2A). Compared to 10 μm melatonin treatment, 3,803 upregulated and 3,244 downregulated DEGs were detected in the axillary buds of plants treated with 1 μm melatonin (Supplementary Figure S2B). However, more DEGs were identified in the axillary buds than basal parts when exposed to the above treatments (Supplementary Figure S1B). For basal parts, 698 upregulated and 813 downregulated DEGs were detected upon treatment with 0.1 μm melatonin compared to the treatment with 1 μm melatonin (Supplementary Figure S3A), and 1,066 upregulated and 946 downregulated DEGs were detected upon treatment with 1 μm melatonin compared to the treatment with 10 μm melatonin (Supplementary Figure S3B), indicating that melatonin has a greater effect on gene expression in rice axillary buds than that in the basal part for axillary buds outgrowth.

Additionally, 2,365 DEGs were upregulated whereas 2,149 DEG were downregulated in the axillary buds of plants exposed to 1.5 mm Lys + 0.5 mm Arg treatment compared to 0 μm melatonin treatment (Supplementary Figures S4A, S5A). Compared to 1.5 mm Lys + 0.5 mm Arg + 1 μm melatonin treatment, 2,107 DEGs were upregulated whereas 2,705 DEGs were downregulated in the axillary buds of plants exposed to 1.5 mm Lys + 0.5 mm Arg treatment (Supplementary Figures S4A, S5B). In the basal parts, 1913 (1,074 upregulated and 839 downregulated) and 465 (298 upregulated and 167 downregulated) DEGs were detected upon 1.5 mm Lys + 0.5 mm Arg treatment compared to 0 μm melatonin (Supplementary Figures S4B, S6A) and 1.5 mm Lys + 0.5 mm Arg + 1 μm melatonin treatments (Supplementary Figures S4B, S6B), respectively, indicating that DEGs in axillary bud was higher than that in basal part under amino acids Lys + Arg treatments.

### DEGs in the Axillary Buds and Basal Parts of Plants Grown Under Different Melatonin and Amino Acid Concentrations

**Figure 2:**
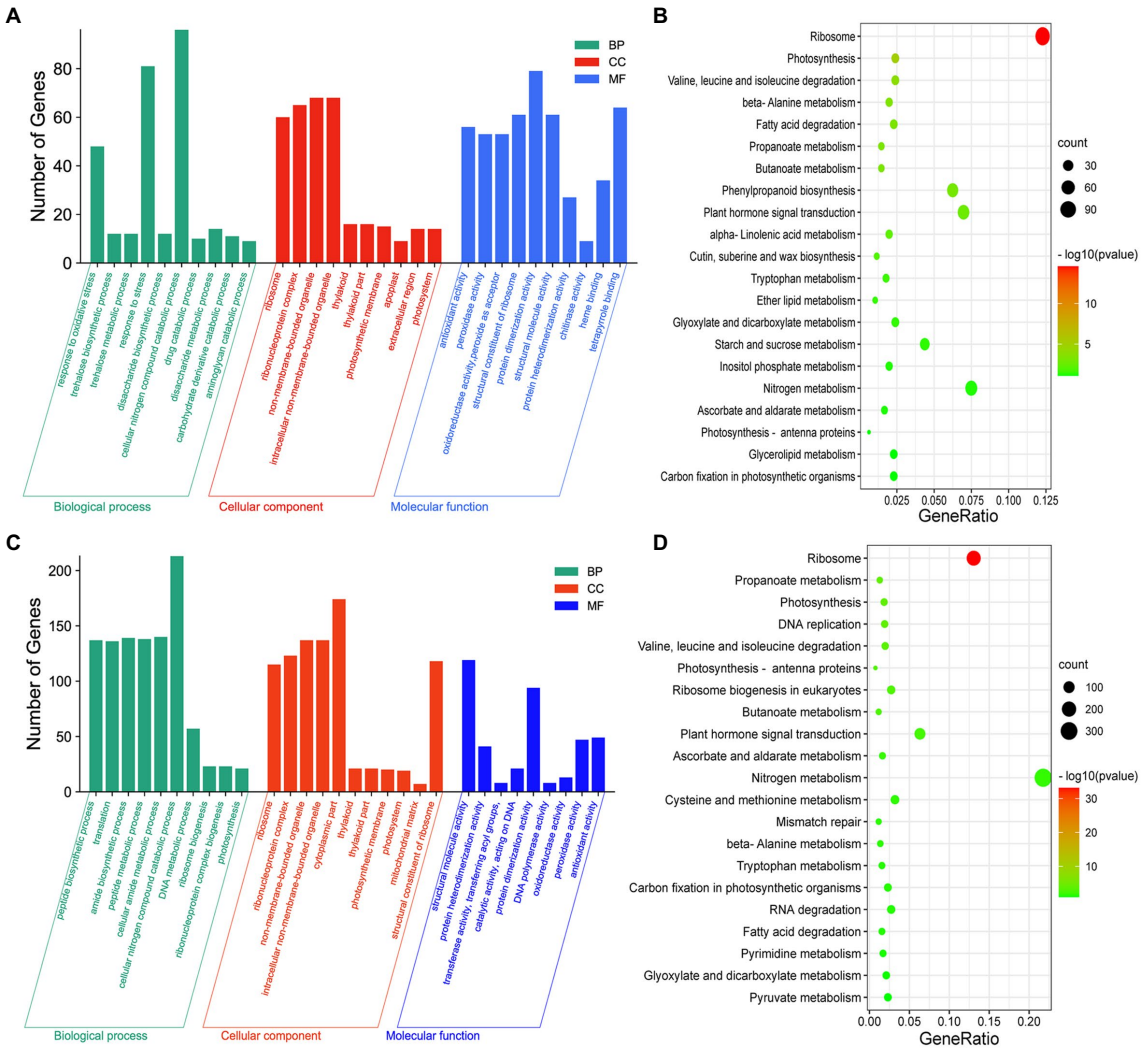
Analysis of differential expression genes (DEGs) with different expression patterns in axillary bud under different concentrations of melatonin treatments. The GO enrichment **(A)** and KEGG pathway enrichment **(B)** analysis of DEGs at 0.1 μm melatonin compared to 1 μm melatonin. The GO enrichment **(C)** and KEGG pathway enrichment **(D)** analysis of DEGs at 1 μm melatonin compared to 10 μm melatonin. Y-axis indicates the number of GO annotated genes, and X-axis indicates the processes/components in different biological processes, cellular components, and molecular functions **(A,C)**. Y-axis indicates KEGG pathway, and X-axis indicates the ratio of the number of enriched genes to the number of annotated genes in the pathway **(B,D)**. The color of the dot represents value of *p*, and the size of the dot represents the number of DEGs mapped to the referent pathway.

To investigate the processes associated with axillary bud outgrowth under different melatonin concentrations, we conducted GO enrichment analysis for each treatment. The genes responsive to 0.1–1 μm melatonin in the axillary buds (Figure 2A) and basal parts (Supplementary Figure S7A) were primarily involved in cellular carbohydrate metabolism and oxidative stress responses. In addition, KEGG analysis indicated that these DEGs were mainly enriched in nitrogen metabolism, plant–pathogen interactions, and phytohormone signal transduction pathways in axillary buds (Figure 2B) and basal parts (Supplementary Figure S7B) of plants exposed to 0.1 μm and 1 μm melatonin treatments. The genes responsive to 1–10 μm melatonin in axillary buds (Figure 2C) and basal parts (Supplementary Figure S7C) were primarily involved in cellular nitrogen compound catabolism, cellular amide metabolism, and amide biosynthesis, and these DEGs were mainly enriched in nitrogen metabolism and phytohormone signal transduction in axillary buds (Figure 2D) and carbon metabolism and amino acid biosynthesis in basal parts (Supplementary Figure S7D). These results indicate that nitrogen metabolism and phytohormone signal transduction pathways play important roles in response to melatonin to promote or inhibit axillary bud outgrowth.

**Figure 3:**
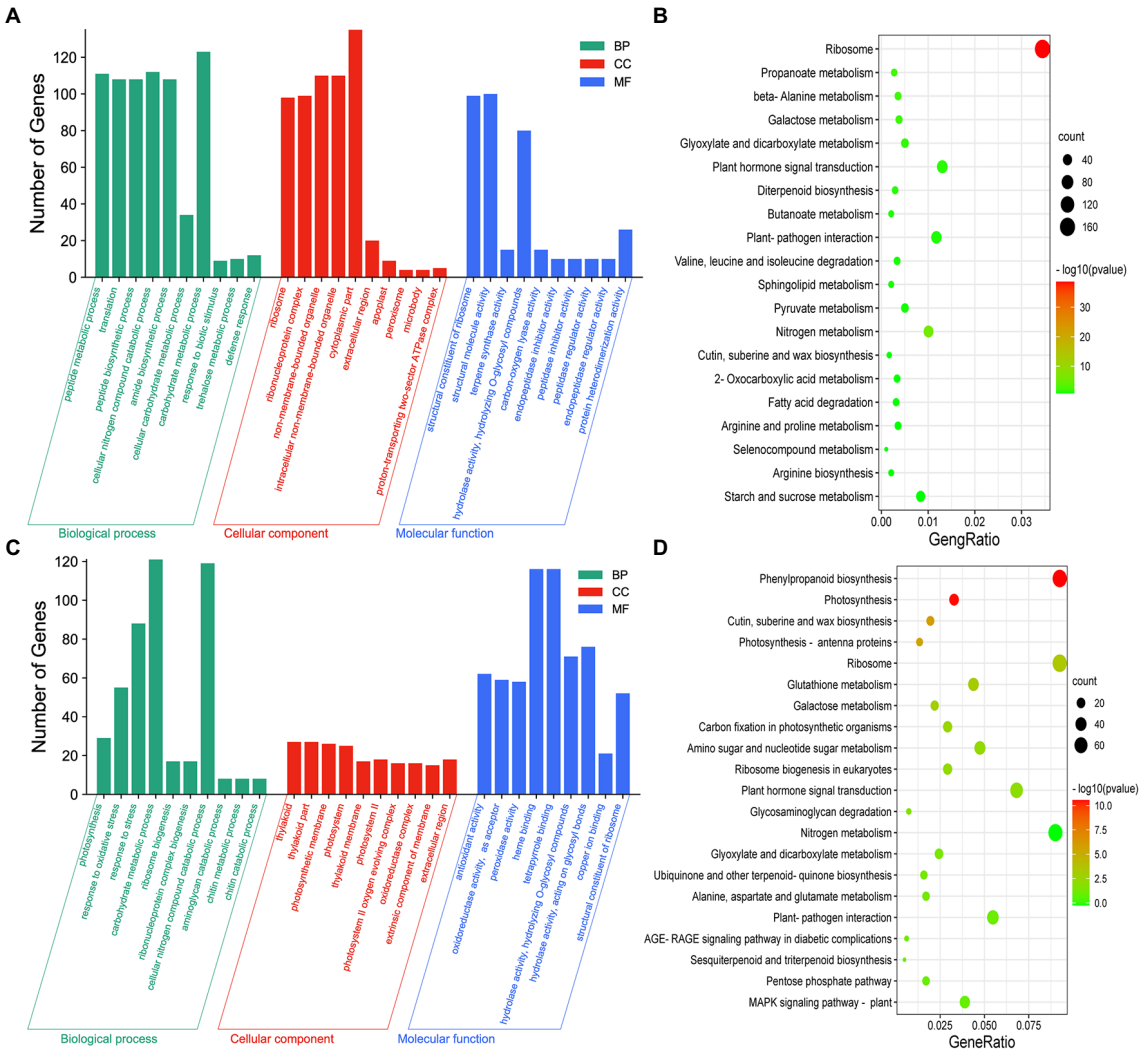
Analysis of differential expression genes (DEGs) with different expression patterns in axillary bud under amino acids related treatments. The GO enrichment **(A)** and KEGG pathway enrichment **(B)** analysis of DEGs at 0 μm melatonin compared to 1.5 mm Lys + 0.5 mm Arg. The GO enrichment **(C)** and KEGG pathway enrichment **(D)** analysis of DEGs at 1.5 mm Lys + 0.5 mm Arg compared to 1.5 mm Lys + 0.5 mm Arg + 1 μm melatonin. Y-axis indicates the number of GO annotated genes, and X-axis indicates the processes/components in different biological processes, cellular components, and molecular functions **(A,C)**. Y-axis indicates KEGG pathway, and X-axis indicates the ratio of the number of enriched genes to the number of annotated genes in the pathway **(B,D)**. The color of the dot represents value of *p*, and the size of the dot represents the number of DEGs mapped to the referent pathway.

To investigate the mechanism underlying the inhibition of axillary bud elongation by Lys and Arg in rice, we conducted GO enrichment analysis of axillary buds of plants exposed to 1.5 mm Lys + 0.5 mm Arg and the control. DEGs in axillary buds (Figure 3A) and basal parts (Supplementary Figure S8A) of plants exposed to 1.5 mm Lys + 0.5 mm Arg treatment were mainly involved in cellular carbohydrate metabolism and amide biosynthesis, and were mainly enriched in nitrogen metabolism, plant–pathogen interactions, and phytohormone signal transduction pathways (Figure 3B; Supplementary Figure S8B). In addition, compared to 1.5 mm Lys + 0.5 mm Arg treatment, DEGs in the axillary buds (Figure 3C) and basal parts (Supplementary Figure S8C) of plants exposed to 1.5 mm Lys + 0.5 mm Arg + 1 μm melatonin were primarily involved in cellular nitrogen compound catabolism, carbohydrate metabolism, and stress responses, and were enriched in nitrogen metabolism, plant–pathogen interactions, photosynthesis, and phytohormone signal transduction pathways in axillary buds (Figure 3D) and photosynthesis, nitrogen metabolism, and carbon metabolism in basal parts (Supplementary Figure S8D). These results indicate that nitrogen metabolism, plant–pathogen interactions, and phytohormone signal transduction pathways play important roles in response to amino acids Lys + Arg alone or amino acids Lys + Arg together with melatonin to promote or inhibit axillary bud outgrowth.

### Expression Profiles of Genes Related to Nitrogen Metabolism, Photosynthesis, and Stress Response Pathways

**Figure 4:**
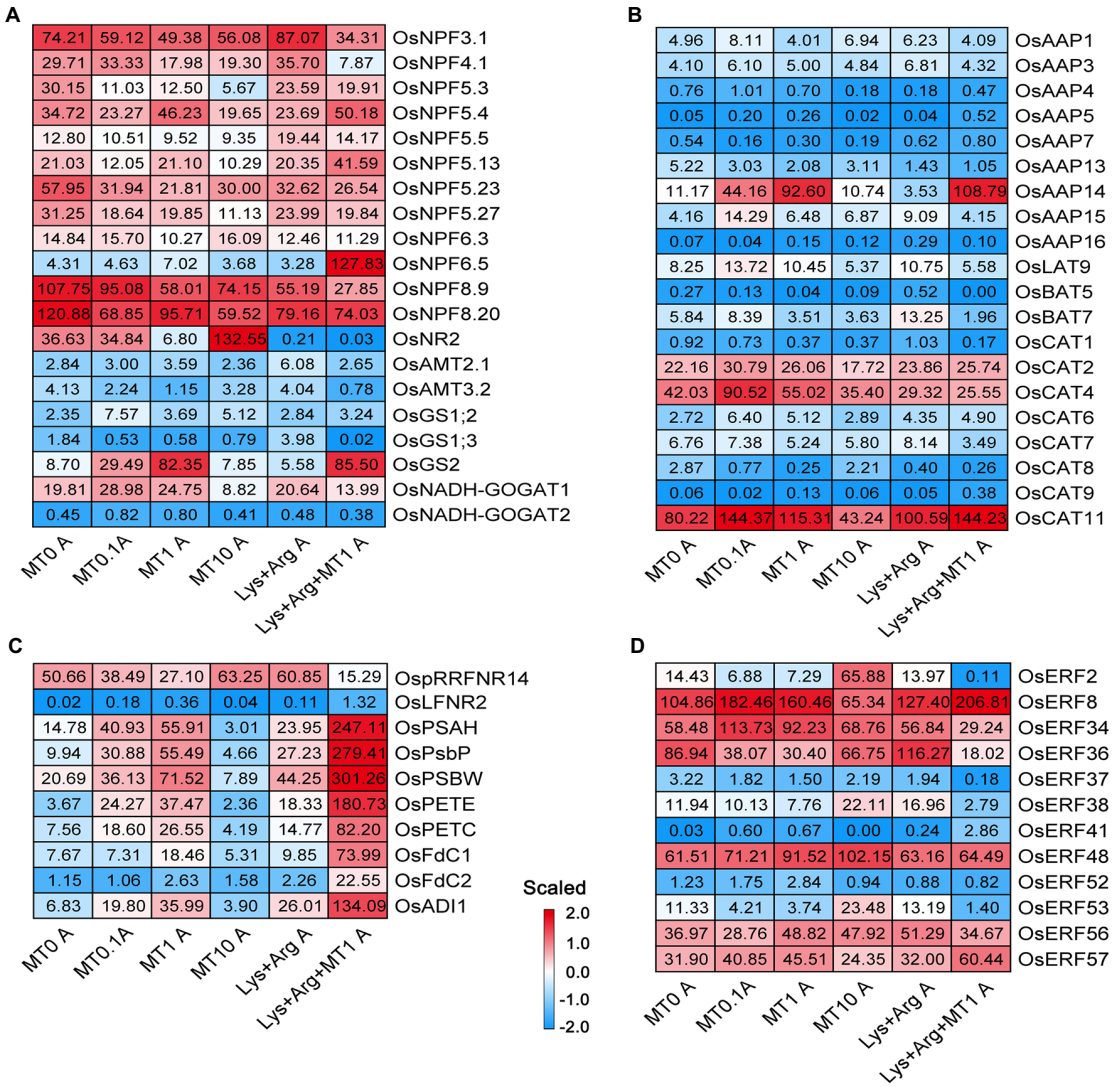
The expression of genes involved in nitrogen metabolism, photosynthesis and stress in axillary bud under different concentrations of melatonin and amino acids. Heatmaps display the expression patterns of genes involved in nitrate and ammonium transport and assimilation **(A)**, amino acid transport **(B)**, photosynthesis **(C)** and stress **(D)**. Red and blue represent the highest and lowest level of expression. MT represents melatonin, Lys represents lysine, and Arg represents arginine. A at the bottom of each graph represents axillary bud, and B at the bottom of each graph represents basal part. MT0, MT0.1, MT1 and MT10 represents melatonin at 0 μm, 0.1 μm, 1 μm and 10 μm, respectively.

Based on the enriched processes associated with nitrogen metabolism, photosynthesis, stress response, and phytohormone pathways in the axillary buds and basal parts, we inferred that these processes predominantly determined the growth rate of axillary buds. Therefore, we analyzed the expression profiles of genes involved in nitrate, ammonium, and amino acid transport and assimilation in axillary buds using a heatmap (Figures 4A,B). Glutamine synthetase gene *OsGS2* (Figure 4A) and amino acid transporter gene *OsAAP14* (Figure 4B) were highly expressed upon treatment with 1 μm melatonin and 1.5 mm Lys + 0.5 mm Arg + 1 μm melatonin, indicating that nitrogen assimilation and amino acid transport were activated by 1 μm melatonin to promote axillary bud growth. The expression of *OsGS2* and *OsAAP14* was strongly inhibited by 10 μm melatonin and 1.5 mm Lys + 0.5 mm Arg (Figures 4A,B). Besides, the expression of *OsCAT11* was also inhibited by 10 μm melatonin (Figure 4B). In addition, the expression of *OsNPF5.4* was increased under low and moderate concentrations of melatonin, whereas *OsNPF6.5* was significantly expressed only when the amino acids were complemented with melatonin (Figure 4A), indicating that melatonin could greatly promote nitrogen transport and assimilation under the high concentration nitrogen stress. However, the ammonium transporter gene was not remarkably expressed upon melatonin treatment (Figure 4A). In addition, in the basal parts, there were no significant differences in the expression of the genes involved in nitrogen metabolism among different melatonin and amino acid treatments (Supplementary Figure S9). These results suggest that the induction of nitrogen assimilation gene *OsGS2* and amino acid transporter gene *OsAAP14* by melatonin was critical for melatonin to promote axillary bud elongation.

In the photosynthetic pathway, the expression of *OsPSAH*, *OsPsbP*, *OsPSBW*, *OsPETE*, *OsPETC*, *OsFdC1*, *OsFdC2*, and *OsADI1* was higher in axillary buds upon treatment with 1 μm melatonin compared to treatment with 0 μm melatonin (Figure 4C). However, the expression of these genes was inhibited by 10 μm melatonin, significantly increased upon treatment with 1.5 mm Lys + 0.5 mm Arg + 1 μm melatonin (Figure 4C), and not inhibited upon treatment with 1.5 mm Lys + 0.5 mm Arg (Figure 4C). This suggests that ferredoxin-associated genes were involved in the regulation rice axillary bud elongation by melatonin, but not in elongation inhibition by Lys and Arg. Furthermore, the expression levels of some genes with unknown functions in the photosynthetic pathway were higher in axillary buds under 1 μm melatonin and 1.5 mm Lys + 0.5 mm Arg + 1 μm melatonin treatments compared to other treatments (Supplementary Figure S10A).

The expression of some genes with unknown functions in the carbon metabolic pathway was higher in axillary buds under 1.5 mm Lys + 0.5 mm Arg + 1 μm melatonin treatment compared to other treatments (Supplementary Figures S10B,C). However, there was no significant difference in the expression of these genes among different treatments in the basal parts (Supplementary Figures S11A–C). In the phenylpropanoid biosynthesis pathway, the expression of some genes was higher in axillary buds (Supplementary Figures S10D–F) and basal parts (Supplementary Figures S11D–F) under 10 μm melatonin treatment compared to other treatments. In the stress response pathway, some ethylene response factor genes were also differentially expressed; however, their expression was neither induced nor inhibited by melatonin (Figure 4D), indicating that melatonin did not directly regulate rice axillary bud elongation. Additionally, only the expression of *OsCML10*, *OsCML15*, and *OsCML31* of the calmodulin family was higher in both axillary buds and basal parts under 1 μm melatonin treatment (Supplementary Figure S12).

### Expression Profiles of Genes Related to Phytohormone Pathways

**Figure 5:**
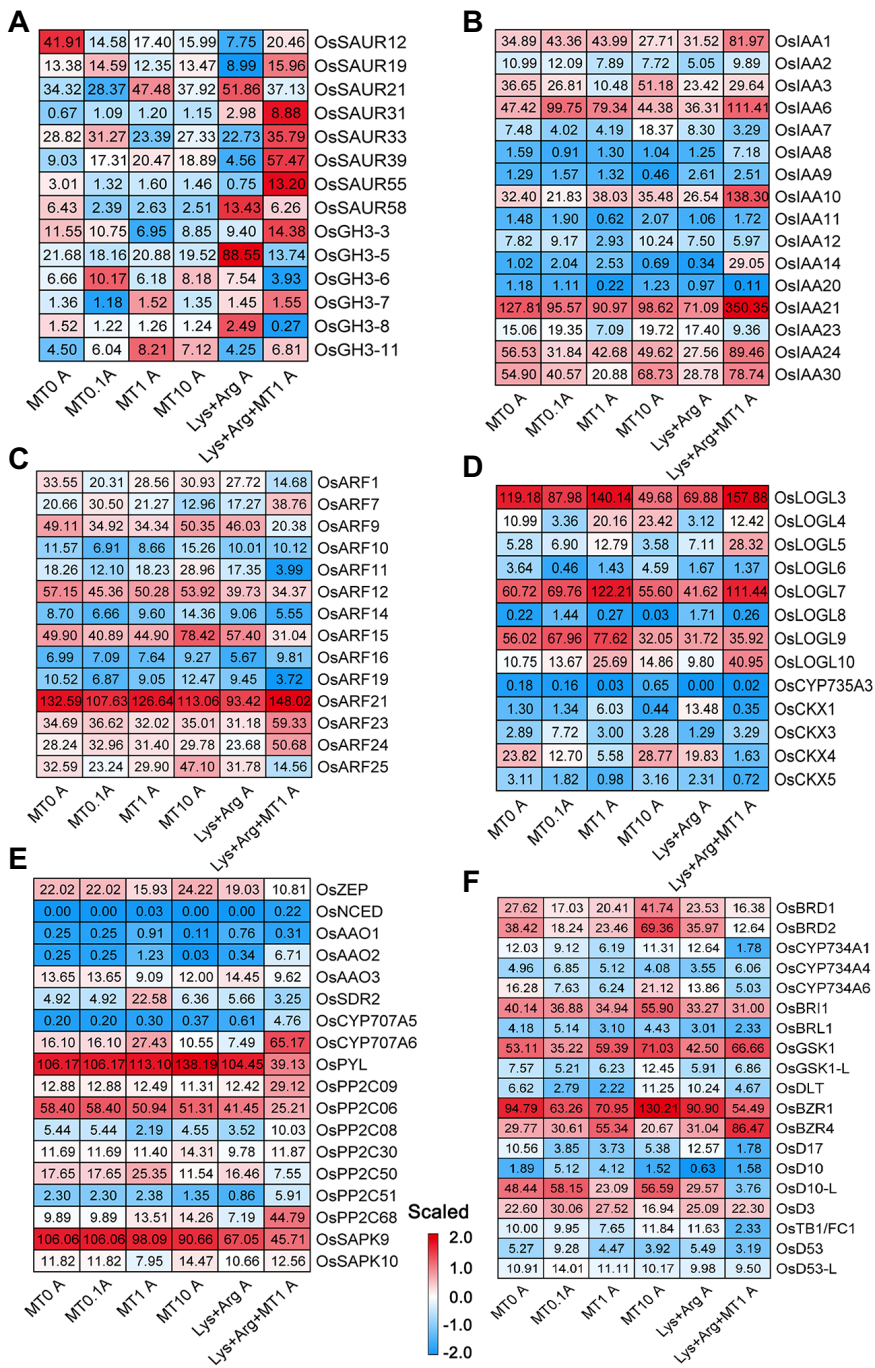
The expression of genes involved in plant hormone signal transduction in axillary bud under different concentrations of melatonin and amino acids. Heatmaps display the expression patterns of genes involved in auxin **(A–C)**, cytokinin **(D)**, abscisic acid **(E)**, ethylene and strigolactone **(F)**. Red and blue represent the highest and lowest level of expression. MT represents melatonin, Lys represents lysine, and Arg represents arginine. A at the bottom of each graph represents axillary bud, and B at the bottom of each graph represents basal part. MT0, MT0.1, MT1 and MT10 represents melatonin at 0 μm, 0.1 μm, 1 μm and 10 μm, respectively.

To determine the functions of phytohormones involved in the axillary bud outgrowth of plants exposed to different melatonin and amino acid treatments, we analyzed the expression profiles of genes associated with phytohormones [auxin, CKs, abscisic acid (ABA), BRs and SLs]. The expression of some auxin-responsive SMALL AUXIN UPREGULATED RNA and Aux/IAA gene family members increased when treated with 1.5 mm Lys + 0.5 mm Arg + 1 μm melatonin compared to other treatments (Figures 5A,B). The expression of AUXIN RESPONSE FACTOR (ARF) gene family members *OsARF15* and *OsARF25* increased in the plants exposed to 10 μm melatonin, whereas that of *OsARF1* decreased in plants treated with 1.5 mm Lys + 0.5 mm Arg + 1 μm melatonin compared to other treatments (Figure 5C). Previous reports have shown that *OsARF1* (Attia et al., 2008) and *OsARF25* (Li et al., 2020) exhibit negative regulatory effects on rice tillers, which indicates that some ARF members involved in the auxin pathway are also involved in the regulation of axillary bud outgrowth by melatonin and amino acids. In the CK pathway, the expression of *OsLOGL5* and *OsLOGL10* increased upon treatment with 1.5 mm Lys + 0.5 mm Arg + 1 μm melatonin, whereas that of *OsCKX4* was only induced upon treatment with 10 μm melatonin compared to other treatments (Figure 5D). Previous reports suggest that both *OsLOGL5* (Hou et al., 2021a) and *OsCKX4* (Wang et al., 2022a) regulate the elongation of axillary buds in rice.

In the ABA pathway, only the expression of *OsSDR2* and *OsPP2C50* was induced in 1 μm melatonin treatment compared to other treatments (Figures 5E,F), whereas the expression of *OsCYP707A6*, *OsPP2C09*, *OsPP2C68*, and *OsBZIP63* was higher in 1.5 mm Lys + 0.5 mm Arg + 1 μm melatonin treatment (Figures 5E,F). In bZIP transcription factor of ABA pathway, the expression of *OsFBL30, and OsbZIP37* in axillary buds was higher under 1 μm Melatonin, and the expression of *OsBZIP63* was higher in 1.5 mm Lys + 0.5 mm Arg + 1 μm melatonin treatment (Supplementary Figure S13). Reports suggest that *OsPP2C09* regulates drought resistance, growth, and development in rice (Miao et al., 2020), indicating that ABA pathway may be involved in the regulation of axillary buds by melatonin and amino acids. In the BRs pathway, the expression of DEGs showed no obvious change under different treatments (Figure 5F). Furthermore, consistent with the role of SLs as inhibitors of axillary bud growth, the expression of SL biosynthesis-related gene *OsD10-L* and signaling-related gene *OsTB1* was low under 1 μm melatonin and 1.5 mm Lys + 0.5 mm Arg + 1 μm melatonin treatments (Figure 5F). In addition, only the expression of *OsLOGL3* in CK pathway was induced upon 1 μm melatonin and 1.5 mm Lys + 0.5 mm Arg + 1 μm melatonin treatments compared to other treatments in the basal parts (Supplementary Figure S14).

### Melatonin and Basic Amino Acids Regulate the Expression of *OsGS2* and *OsAAP14* in Rice Roots

**Figure 6:**
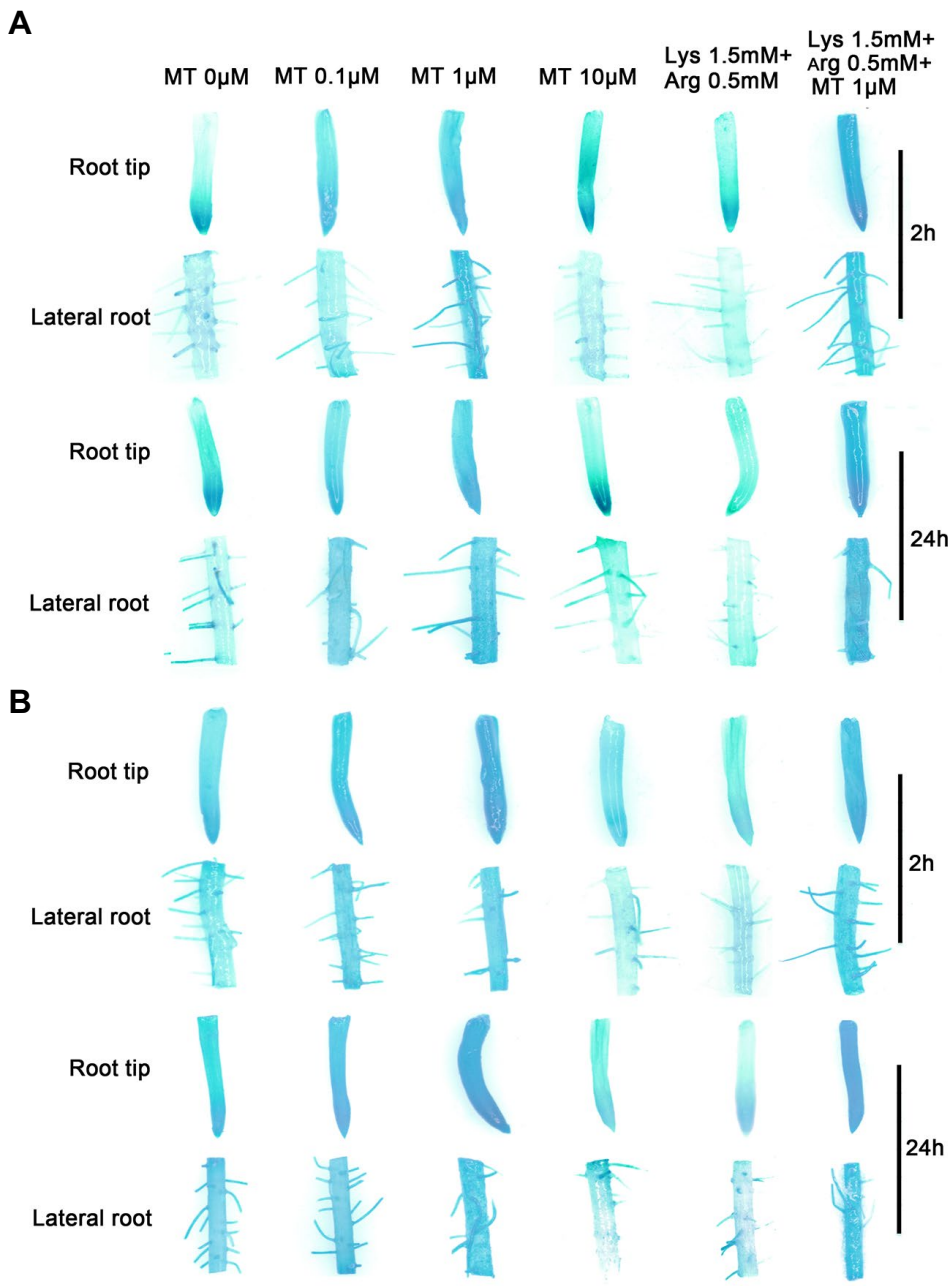
The expression of *OsAAP14* and *OsGS2* is regulated by melatonin and amino acids. Promoter-GUS transgenic seedlings were grown under basic nutrient solutions supplemented with different concentrations of melatonin (0 μm, 0.1 μm, 1 μm, 10 μm), 1.5 mm Lys + 0.5 mm Arg, or 1.5 mm Lys + 0.5 mm Arg + 1 μm melatonin for 2 h and 24 h, respectively. *OsAAP14* promoter-GUS staining in the root tip and lateral root **(A)**. *OsGS2* promoter-GUS staining in the root tip and lateral root **(B)**. MT represents melatonin, Lys represents lysine, and Arg represents arginine.

Since melatonin, Lys, and Arg, which are involved in the nitrogen metabolism pathway, affected the expression of *OsGS2* and *OsAAP14* (Figures 4A,B), *OsGS2* and *OsAAP14* promoter-GUS transgenic lines were perform for experiments. GUS staining and RT-qPCR showed that *OsGS2* was highly expressed in root tip (Supplementary Figures S15A,H), lateral root (Supplementary Figures S15B,H) basal part (Supplementary Figures S15C,H), culm (Supplementary Figures S15D,H) and panicle (Supplementary Figures S15G,H), but was only slightly expressed in leaf sheath (Supplementary Figures S15E,H) and leaf blade (Supplementary Figures S15F,H). Furthermore, we found a similar expression pattern in *OsAAP14* (Supplementary Figure S16). Besides, *OsGS2* and *OsAAP14* promoter-GUS transgenic lines were treated with exogenous melatonin, Lys, and Arg to determine whether they altered the expression of the above mentioned genes (Figure 6). Results showed that the root tip and lateral roots of *OsGS2* (Figure 6A) and *OsAAP14* (Figure 6B) promoter-GUS transgenic rice plants treated with 1 μm melatonin were deeply stained at both 2 h and 24 h compared to other concentrations of melatonin. With the increase in melatonin concentration (0–1 μm), GUS staining gradually deepened, but the intensity of the stain decreased when the concentration exceeded 1 μm (Figure 6). To systematically characterize the regulation of *OsGS2* and *OsAAP14* expression by basic amino acids or their combination with melatonin, GUS staining was performed after treatments with 1.5 mm Lys + 0.5 mm Arg or 1.5 mm Lys + 0.5 mm Arg + 1 μm melatonin. Weak and strong staining was observed at 2 h and 24 h upon 1.5 mm Lys + 0.5 mm Arg and 1.5 mm Lys + 0.5 mm Arg + 1 μm melatonin treatments, respectively (Figure 6). The above results were further determined by RT-qPCR (Supplementary Figure S17).

**Figure 7:**
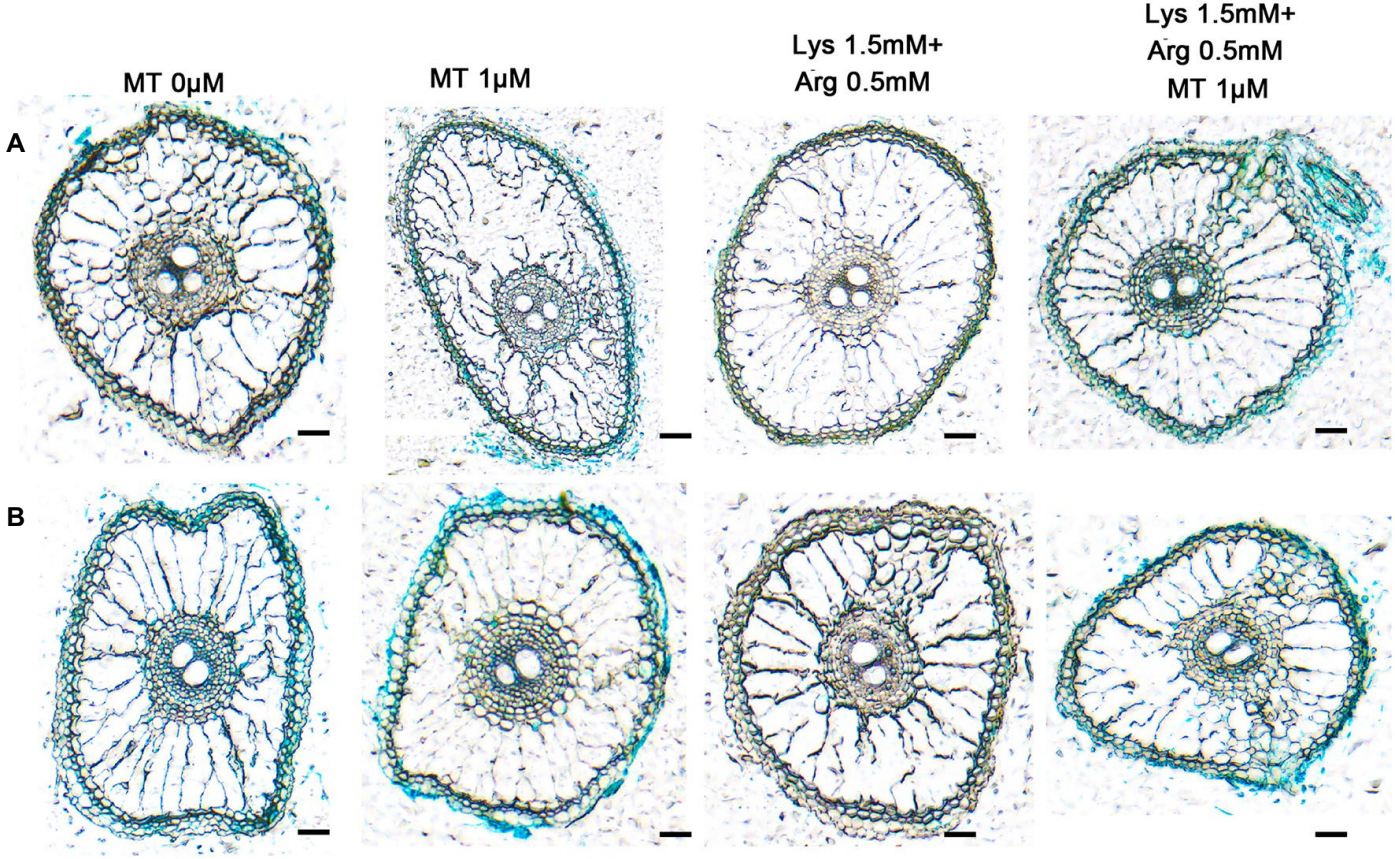
Section analysis of *OsAAP14* and *OsGS2* promoter-GUS transgenic plants regulating by melatonin and amino acids. Promoter-GUS transgenic seedlings were grown under basic nutrient solutions supplemented with different concentrations of melatonin (0 μm, 0.1 μm, 1 μm, 10 μm), 1.5 mm Lys + 0.5 mm Arg, or 1.5 mm Lys + 0.5 mm Arg + 1 μm melatonin. Paraffin sections of the stained roots of *OsAAP14* **(A)** and *OsGS2* **(B)** promoter-GUS transgenic plants. MT represents melatonin, Lys represents lysine, and Arg represents arginine. Scale bar = 40 μm.

In addition, it showed that the axillary bud and basal part of *OsAAP14* (Supplementary Figure S18A) and *OsGS2* (Supplementary Figure S18B) promoter-GUS transgenic rice plants were deeply stained under 1 μm melatonin compared to other concentrations of melatonin. With the increase in melatonin concentration (0–1 μm), GUS staining gradually deepened, but the intensity of the stain decreased when the concentration exceeded 1 μm, and weak and strong staining was observed upon 1.5 mm Lys + 0.5 mm Arg and 1.5 mm Lys + 0.5 mm Arg + 1 μm melatonin treatments, respectively (Supplementary Figures S18A,B). The above results were further determined by RT-qPCR (Supplementary Figures S18C,D). Furthermore, to determine the tissue-specific expression pattern of *OsAAP14* and *OsGS2*, sections of the GUS-stained organs were made (Figure 7). Sectioning confirmed that the expression of *OsAAP14* was observed in the cortex parenchyma and vascular tissue of the root, and GUS activity was abundant in 1 μm melatonin or 1.5 mm Lys + 0.5 mm Arg + 1 μm melatonin treatments compared to other treatments (Figure 7A). Similarly, GUS activity of *OsGS2* was abundant in the cortex parenchyma and vascular tissue of the root especially under 1 μm melatonin or 1.5 mm Lys + 0.5 mm Arg + 1 μm melatonin treatments compared to other treatments (Figure 7B). Overall, these observations show that *OsGS2* and *OsAAP14* were highly expressed in the roots and basal parts in response to melatonin and amino acids.

### Altered Expression of *OsGS2* and *OsAAP14* Influences Axillary Bud Outgrowth in Rice

**Figure 8:**
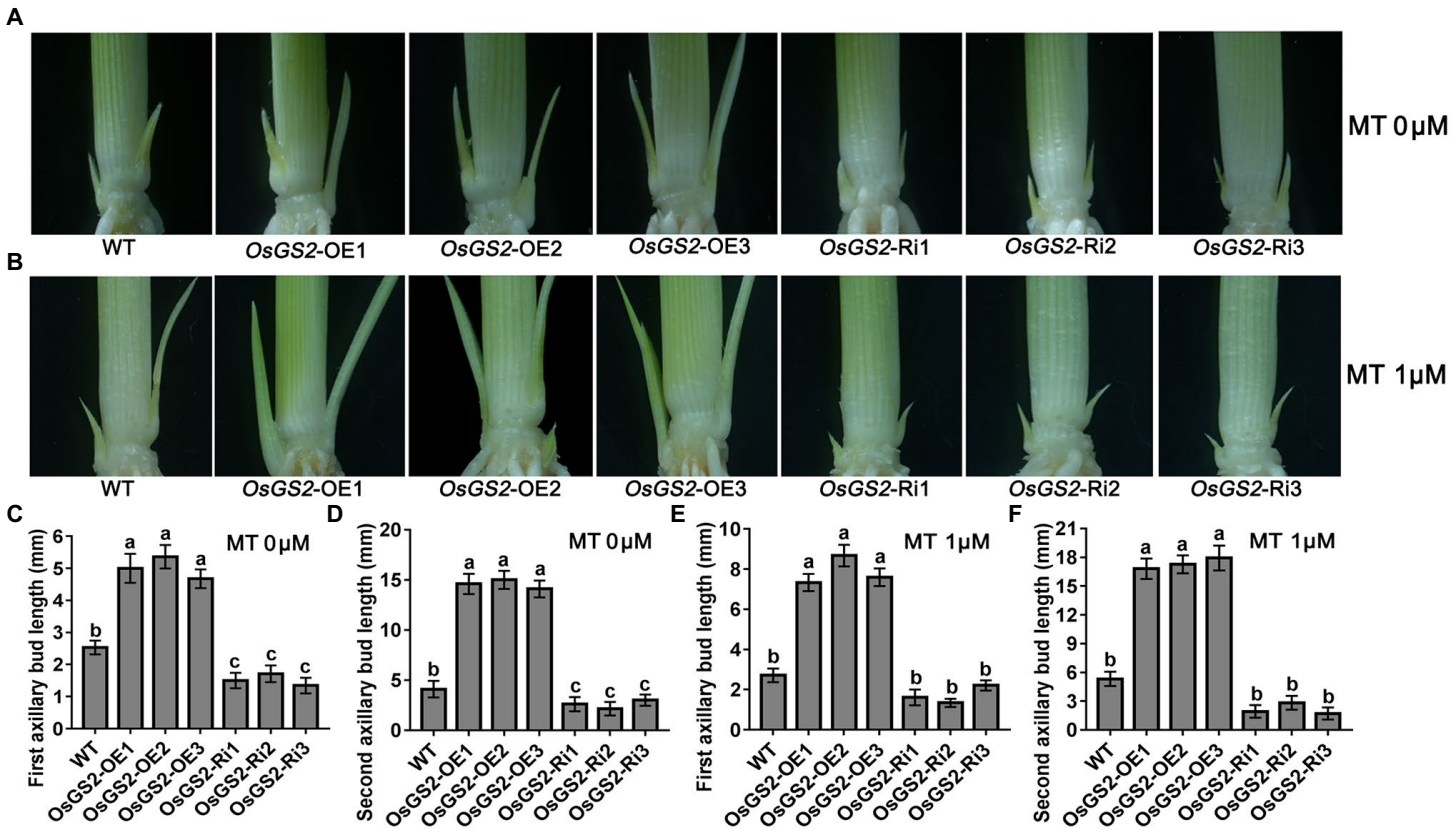
Effect of melatonin on outgrowth axillary bud of *OsGS2* transgenic seedlings. Phenotypes of outgrowth axillary bud with altered expression of *OsGS2* grown under 0 μm melatonin **(A)** and 1 μm melatonin **(B)**. Statistical analysis of the first **(C,E)** and second **(D,F)** axillary bud length in **(A,B)**. Values are means ± SD (*n* > 20), and the significance levels of different lowercase letters were as follows, *p* < 0.05. WT represents wild-type ZH11, OE1-OE3 represent three lines of *OsGS2* over-expressing transgenic plants, and Ri1-Ri3 represent three lines of *OsGS2* RNAi transgenic plants. MT represents melatonin.

**Figure 9:**
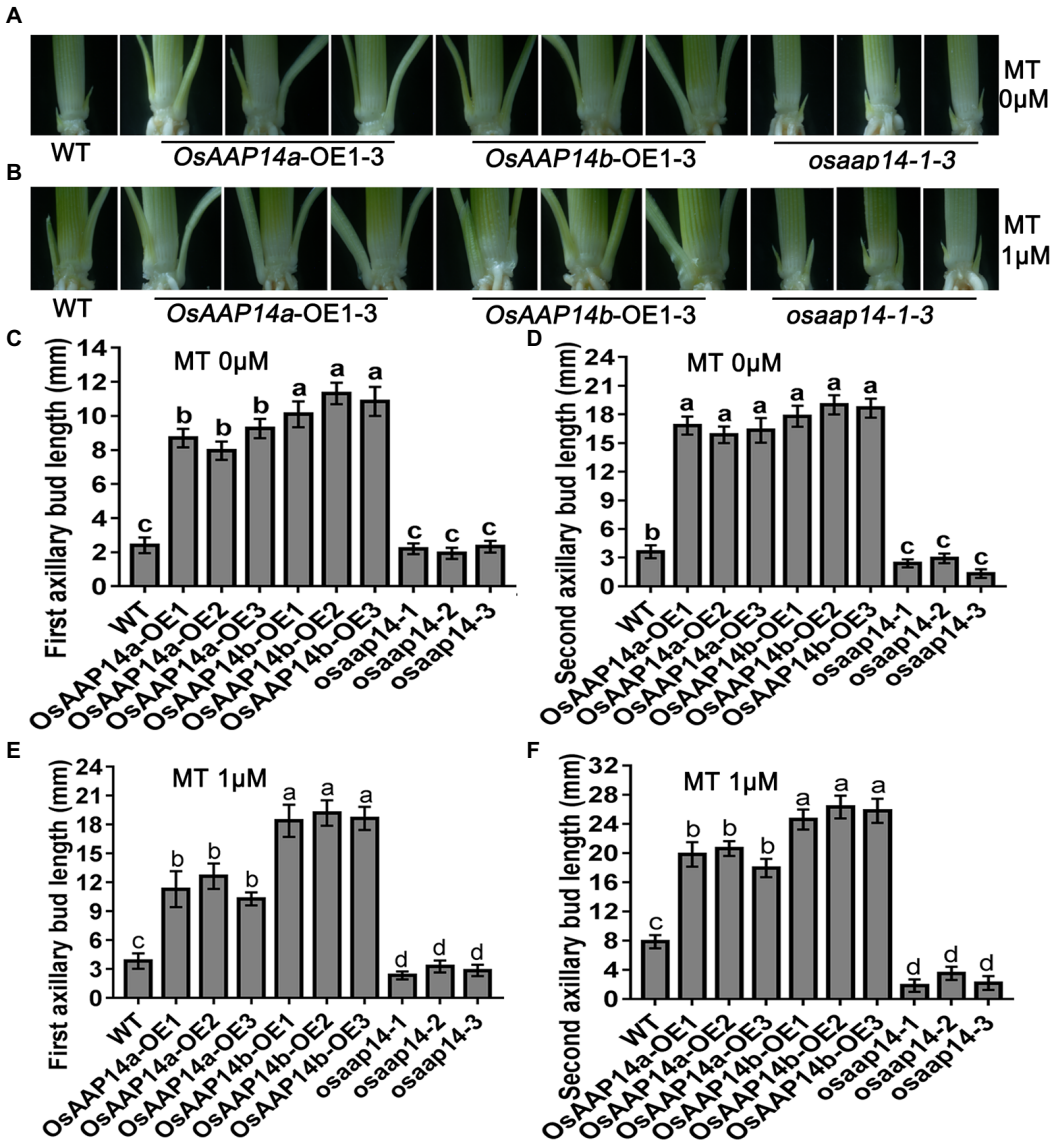
Effect of melatonin on outgrowth axillary bud of *OsAAP14* transgenic seedlings. Phenotypes of outgrowth axillary bud with altered expression of *OsAAP14* grown under 0 μm melatonin **(A)** and 1 μm melatonin **(B)**. Statistical analysis of the first **(C,E)** and second **(D,F)** axillary bud length in **(A,B)**. Values are means ± SD (*n* > 20), and the significance levels of different lowercase letters were as follows, *p* < 0.05. WT represents wild-type ZH11, *OsAAP14a-*OE1-OE3 represent three lines of *OsAAP14* longer splicing variant over-expressing transgenic plants, *OsAAP14b-*OE1-OE3 represent three lines of *OsAAP14* shorter splicing variant over-expressing transgenic plants, and *osaap14-1-3* represent three lines of *OsAAP14* CRISPR mutants. MT represents melatonin.

To further determine the role of *OsGS2* and *OsAAP14* in regulating rice axillary bud outgrowth in response to melatonin and basic amino acid treatments, we generated *OsGS2* OE and RNAi (Ri) lines and *OsAAP14* OE (two splicing variants: *OsAAP14*a-OE and *OsAAP14b*-OE) and CRISPR lines in the *japonica* ZH11 background (Supplementary Figures S19, S20). We found that both the first and second axillary bud length in the *OsGS2* OE line increased when grown in control solution compared to the Ri line and WT (Figures 8A,C,D). Furthermore, both the length of first and second axillary buds of the OE lines and WT significantly increased under 1 μm melatonin treatment, compared to their corresponding OE lines and WT grown in normal solution (Figures 8B,E,F). However, the axillary buds of the *OsGS2* Ri line were short, and there was no difference in the length of axillary buds exposed to melatonin and normal solution (Figure 8). This indicated that the expression of *OsGS2* in the Ri line induced by melatonin was lost, and that melatonin no longer influenced the elongation of axillary buds after the reduction in *OsGS2* expression. Similarly, the first and second bud length in the *OsAAP14a* and *OsAAP14b* OE lines increased when grown in normal solution compared to that of the *OsAAP14* CRISPR line and WT (Figures 9A,C,D). When treated with 1 μm melatonin, the axillary buds of *OsAAP14a* and *OsAAP14b* OE lines and WT further elongated, but the axillary bud length of the *OsAAP14* CRISPR line did not change (Figure 9). Besides, we also found that both variants of *OsAAP14* OE lines enhanced grain yield by increasing tiller and grain number per plant, while the *OsAAP14* CRISPR line exhibited significantly reduced tiller number and grain yield (Supplementary Figure S21). In addition, it was showed that the contents of total free amino acids and total chlorophyll were significantly increased both in *OsAAP14* OE lines and *OsGS2* OE lines compared with WT, while significantly decreased in *OsAAP14* CRISPR lines and *OsGS2* Ri lines compared with WT (Supplementary Figure S22).

To investigate why the effect of 1.5 mm Lys, 0.5 mm Arg and 1 μm melatonin on axillary bud elongation was stronger than that of 1 μm melatonin alone, seedlings amino acid-uptake assay was performed under 1 μm melatonin. Stronger fluorescence signals were detected in the *OsAAP14* OEb lines cultured with 0.5 mm Arg-FITC and 0.5 mm Lys-FITC for 2 h or 6 h than those of the ZH11 and OEa lines, and the FITC signal was weaker in Ri lines than in ZH11 (Supplementary Figures S23, S24). Similarly, Stronger fluorescence signals were detected in the *OsGS2* OE lines cultured with 0.5 mm Arg-FITC, and 0.5 mm Lys-FITC for 2 h or 6 h than those of the ZH11, and the FITC signal was weaker in Ri lines than in ZH11 (Supplementary Figures S23, S24). These results suggest that *OsGS2* and *OsAAP14* play an important role in response to melatonin and the subsequent regulation of axillary bud outgrowth, tillering, and grain yield.

## Discussion

### Exogenous Low Melatonin Promotes Axillary Bud Outgrowth in Rice

Melatonin promotes coleoptile growth in four monocot species including canary grass, wheat, barley, and oat (Hernandez-Ruiz et al., 2005), and increases root growth in *Brassica juncea* (Chen et al., 2009). And it was found that melatonin might function as an auxin to promote vegetative growth (Kolář and Macháčková, 2005). Recently, the identification of the melatonin receptor CAND2/PMTR1 (Wei et al., 2018) established that the phytohormone melatonin plays an important role in plant growth, development, and stress resistance. However, whether melatonin affects axillary bud elongation and tillering in rice still remains unclear. In this study, we found that low concentrations of melatonin (0–1 μm) promoted the elongation of the first and second axillary buds in rice, but higher melatonin concentrations (10–1,000 μm) inhibited the elongation of these buds (Figure 1). The previous study reported that lateral root growth is stimulated at low melatonin levels, while lateral root growth is inhibited at higher melatonin levels (Park, 2011). Furthermore, a low concentration promotes adventitious root regeneration but a growth inhibitory effect at high concentrations, and the negative effect of the high melatonin concentration could be due to antagonism between melatonin and Ca^2+^-calmodulin (Sarropoulou et al., 2012). Thus, our results demonstrate that exogenous melatonin also promotes or inhibits axillary bud outgrowth in rice.

Similarly, previous study reported that 0.1 mm melatonin has a stimulatory effect on root growth of *B. juncea*, while 100 mm is inhibitory, and the effect on root growth and endogenous indole-3-acetic acid (IAA) levels determined (Chen et al., 2009). This study showed that the expression of some auxin-responsive and Aux/IAA gene family members increased when treated with 1.5 mm Lys + 0.5 mm Arg + 1 μm melatonin compared to other treatments (Figure 5). This indicates that there is a link between melatonin and auxin in growth regulation. Besides, the inhibitory effect of SLs was detectable at GR24 (a synthetic SL analogue) concentrations as low as 10 nM, and axillary bud outgrowth was approximately completely inhibited at 1 μm GR24 (Umehara et al., 2008). Furthermore, SLs inhibited axillary bud outgrowth in rice by altering the tillering inhibition pathway through increasing the expression of SL signaling-related gene *OsTB1* (Fang et al., 2020). In our study, the expression of *OsTB1* was low under 1 μm melatonin alone or 1.5 mm Lys + 0.5 mm Arg + 1 μm melatonin treatments (Figure 5). This suggests that melatonin may affect rice axillary buds through the expression of *OsTB1* in SL pathway. In addition, BRs play a significant role in promoting rice tillering; 1 μm CS (a synthetic BR analogue) strongly promoted axillary bud outgrowth and significantly enhanced tillering in rice (Fang et al., 2020). However, different melatonin concentrations has no significant effect on the expression of BRs related genes (Figure 5). Therefore, how Auxin and SL pathways interact with melatonin in regulating rice axillary bud elongation, needs to be further studied.

### Melatonin Relieves the Inhibition of Axillary Bud Outgrowth Under High Concentration of Lys and Arg

Amino acids are crucial for basal metabolism as they participate in protein synthesis and modulate plant growth and development (Yadav et al., 2015). It has been reported that Lys can inhibit mitotic activity in the root apical meristem, and that exogenous Lys can reduce the length of the main root of *Arabidopsis* (Yang et al., 2014). Recently, it has been shown that low concentrations of Lys and Arg promoted the elongation of rice buds, but their high concentrations inhibited bud elongation (Lu et al., 2018). In this study, the addition of high concentrations of Lys and Arg inhibited the elongation of first and second axillary buds in rice (Figure 1). One possible explanation is that Lys and Arg can influence antioxidant enzymes activities and photosynthetic pathway by initiating the ROS accumulation and nitric oxide signaling pathway (Wei et al., 2021). Our results also showed that nitrogen metabolism, stress, and photosynthetic pathways play important roles in response to amino acids Lys and Arg in axillary buds. However, the complementation of these amino acids were with 1 μm melatonin not only alleviated their inhibitory effect on rice axillary buds but also considerably promoted axillary bud elongation (Figure 1). This may be due to the dual role of melatonin in stress resistance and growth promotion. One previous study reported that melatonin enhances plant growth and abiotic stress tolerance in soybean plants (Wei et al., 2015). Transcriptome analysis revealed that melatonin may exert its functions mainly through regulation of photosynthesis, the cell cycle, DNA replication, starch/sucrose metabolism, and lipid biosynthesis (Wei et al., 2015).

Besides, it has been reported that the addition of melatonin lessens the amount of oxidative damage brought on by salinity, perhaps by directly scavenging H_2_O_2_ or enhancing the activities of antioxidative enzymes such as ascorbate peroxidase, catalase, and peroxidase (Li et al., 2012). And 0.1 μm melatonin significantly alleviates growth inhibition and enables plants to maintain an improved photosynthetic capacity in *Malus hupehensis Rehd* (Li et al., 2012). In addition, melatonin promotes seed germination under high salinity by regulating antioxidant systems, ABA and GA4 interaction in cucumber (Zhang et al., 2014). Our results showed that the expression of *OsPSAH*, *OsPsbP*, *OsPSBW*, *OsPETE*, *OsPETC*, *OsFdC1*, *OsFdC2*, and *OsADI1* in photosynthetic pathway was higher in axillary buds upon treatment with 1.5 mm Lys + 0.5 mm Arg + 1 μm melatonin compared to other treatments (Figure 4). Therefore, it was demonstrated that melatonin promoted axillary bud outgrowth and tillering in rice exposed to high concentrations of basic amino acids, indicating that melatonin can alleviate the inhibition of rice axillary bud elongation under nitrogen stress, such as high concentrations of amino acids Lys and Arg.

### Key Genes of Nitrogen Metabolism That Respond to Changes in Melatonin and Amino Acids to Regulate Tillering in Rice

Previously, pathway enrichment analysis indicated that eight pathways were over-represented among differentially expressed genes between control and melatonin-treated bermudagrass plants, including N metabolism, major carbohydrate metabolism, TCA/org transformation, transport, hormone metabolism, metal handling, redox, and secondary metabolism (Shi et al., 2015). In this study, we found that the expression of nitrate transporter gene *OsNPF6.5*, the glutamine synthetase gene *OsGS2* and amino acid transporter gene *OsAAP14* involved in nitrogen metabolism was regulated by different concentrations of melatonin, while the expression of these genes was inhibited by basic amino acid treatment (Figure 4). If melatonin was added with basic amino acids, the expression of these genes notably increased (Figure 4). It was reported that *OsNPF6.5* (*OsNRT1.1B*) indica variant enhances nitrate uptake, tiller number and nitrogen use efficiency in rice (Hu et al., 2015). Recently, glutamine synthetase *OsGS2* has been shown to respond to different nitrogen concentrations, and regulate the elongation of axillary buds and tiller number in rice (Wang et al., 2020). Besides, the previous study indicated that another glutamine synthetase gene *OsGS1.2* could mediate axillary buds outgrowth and tiller number in rice by regulating CK pathway (Yamaya and Kusano, 2014). In addition, other amino acid transporter genes, such as *OsAAP1* and *OsAAP4*, positively regulate rice axillary bud outgrowth and tillering by promoting the transport of neutral amino acids (Ji et al., 2020; Fang et al., 2021). Therefore, melatonin could greatly promote nitrogen transport and assimilation through relieving the high concentration nitrogen inhibition. We also found that the grain yield was enhanced in *OsAAP14* OE lines by increasing tiller and grain number per plant compared with WT (Supplementary Figure S21), and the contents of total free amino acids and total chlorophyll were also significantly increased both in *OsAAP14* OE lines and *OsGS2* OE lines compared with WT (Supplementary Figure S22). This indicates that *OsNPF6.5*, *OsGS2* and *OsAAP14* are key melatonin- and basic amino acid-responsive genes of nitrogen metabolism that regulate axillary bud outgrowth in rice. Overall, the present study provides insights into axillary bud outgrowth in plants, and how melatonin can help improve rice grain yield, especially under nitrogen stress.

## Concluding Remarks

In this study, we found that different concentrations of melatonin influenced axillary bud outgrowth in rice, and moderate melatonin concentrations alleviated the inhibition of axillary bud outgrowth under high concentrations of Lys and Arg. RNA-seq data indicated that the genes involved in nitrogen metabolism, phytohormone signal transduction, and phenylpropanoid biosynthesis were involved in the elongation of axillary bud outgrowth under different melatonin, Lys, and Arg concentrations. In addition, we indicated that the rice glutamine synthetase gene *OsGS2* and amino acid transporter *OsAAP14*, involved in the nitrogen metabolism pathway, were involved in the regulation of axillary bud outgrowth under different concentration of melatonin, Lys, and Arg. These results elucidate the regulatory mechanism underlying the effect of melatonin on axillary bud outgrowth under different amino acid concentrations, and provide insights into improving rice tillering and grain yield using melatonin under amino acid-rich environments.

## Data Availability Statement

The original contributions presented in the study are included in the article/Supplementary Material, further inquiries can be directed to the corresponding author.

## Author Contributions

ZF designed the study. GY and XW performed the experiments. ZF and GY drafted the manuscript. All authors contributed to the article and approved the submitted version.

## Funding

This research was supported by the Wuhan Science and Technology Project (2020020601012259), the Talent Project from Guizhou Education Department [Qian jiao he KY zi (2021) 024], the Key Cultivation Project of Guizhou University (201903).

## Conflict of Interest

The authors declare that the research was conducted in the absence of any commercial or financial relationships that could be construed as a potential conflict of interest.

## Publisher’s Note

All claims expressed in this article are solely those of the authors and do not necessarily represent those of their affiliated organizations, or those of the publisher, the editors and the reviewers. Any product that may be evaluated in this article, or claim that may be made by its manufacturer, is not guaranteed or endorsed by the publisher.

## Footnotes

^1^ www.omicshare.com
