# Supplementary material for: Melatonin Mediates Axillary Bud Outgrowth by Improving Nitrogen Assimilation and Transport in Rice
Source: Front Plant Sci. 2022 Jul 13;13:900262. doi: 10.3389/fpls.2022.900262 (PMC9326366; doi:10.3389/fpls.2022.900262)
Supplement: Supplementary file 1 [file Data_Sheet_1.docx]

Supplementary Material

# Supplementary Tables

**Supplementary Table 1.** The primers used in our study.

| **Primers used for qPCR** | | |
| --- | --- | --- |
| **Gene** | **Forward sequence (5' - 3')** | **Reverse sequence (5' - 3')** |
| *OsActin* | CGGTGTCATGGTCGGAAT | GCTCGTTGTAGAAGGTGT |
| *OsAAP14a* | CAACGCACCGGGAATCTA | GCAGCAACCCACATAACCA |
| *OsAAP14b* | GTGATGGAACTGTCAAAGGGAA | TGGCGATCTCAGCGTGTC |
| *OsGS2* | TAGTGCTTACCCTTGACCC | AACCTCCTTTCATTTCCTTC |
| **Primers for plasmid construction** | | |
| **Gene** | **Forward sequence (5' - 3')** | **Reverse sequence (5' - 3')** |
| p*OsAAP14-GUS* | TAGGATCCAGGGGTTATCCTTTCGC | ATAAGCTTTGTCAAAGGCTCAGTGAA |
| *OsAAP14a*-OE | ATGAGCTCATGGCGCCGCAGCTGCC | TATCTAGAGCCAAGCCTCTTTCTGAT |
| *OsAAP14b*-OE | ATGAGCTCATGGACGTGCGTAGCGCA | TATCTAGAGCCAAGCCTCTTTCTGAT |
| *OsAAP14*- U6 | CCGTGACGCTCCCTGCGACTCGAGTTTTAGAGCTAGAAATAGCAAGTTA | TCGAGTCGCAGGGAGCGTCACGGAACCTGAGCCTCAGCGCAGC |
| *OsAAP14*- U3 | CCAATTTCCACAAGATGGCGTGGGTTTTAGAGCTAGAAATAGCAAGTTA | CCACGCCATCTTGTGGAAATTGGGCCACGGATCATCTGCACAACTC |
| *OsGS2-GUS* | AGGATCCTTTAAAAATGGTTTCTCAGTTGCC | ATTCTAGATTTCACCCCCTACAGATAATCACC |
| *OsGS2*-OE | TAGAGCTCATGGCGCAGGCGGTGGTGCCGGCG | ATTCTAGATACCTTCAGGGCCAACTTCTTAGC |
| *OsGS2*-Ri | GGGGTACCACTAGTGCGATGCAGTGCCAGGTC | CGGGATCCGAGCTCTGTACTCGGCGATGATCTTGTC |
| **Primers for *OsAAP14* mutant identification** | | |
| **Gene** | **Forward sequence (5' - 3')** | **Reverse sequence (5' - 3')** |
| *OsAAP14* | CGTCAGAGTTCACTTAGGGAGG | TGGCGATCTCAGCGTGTC |

# Supplementary Figures

*
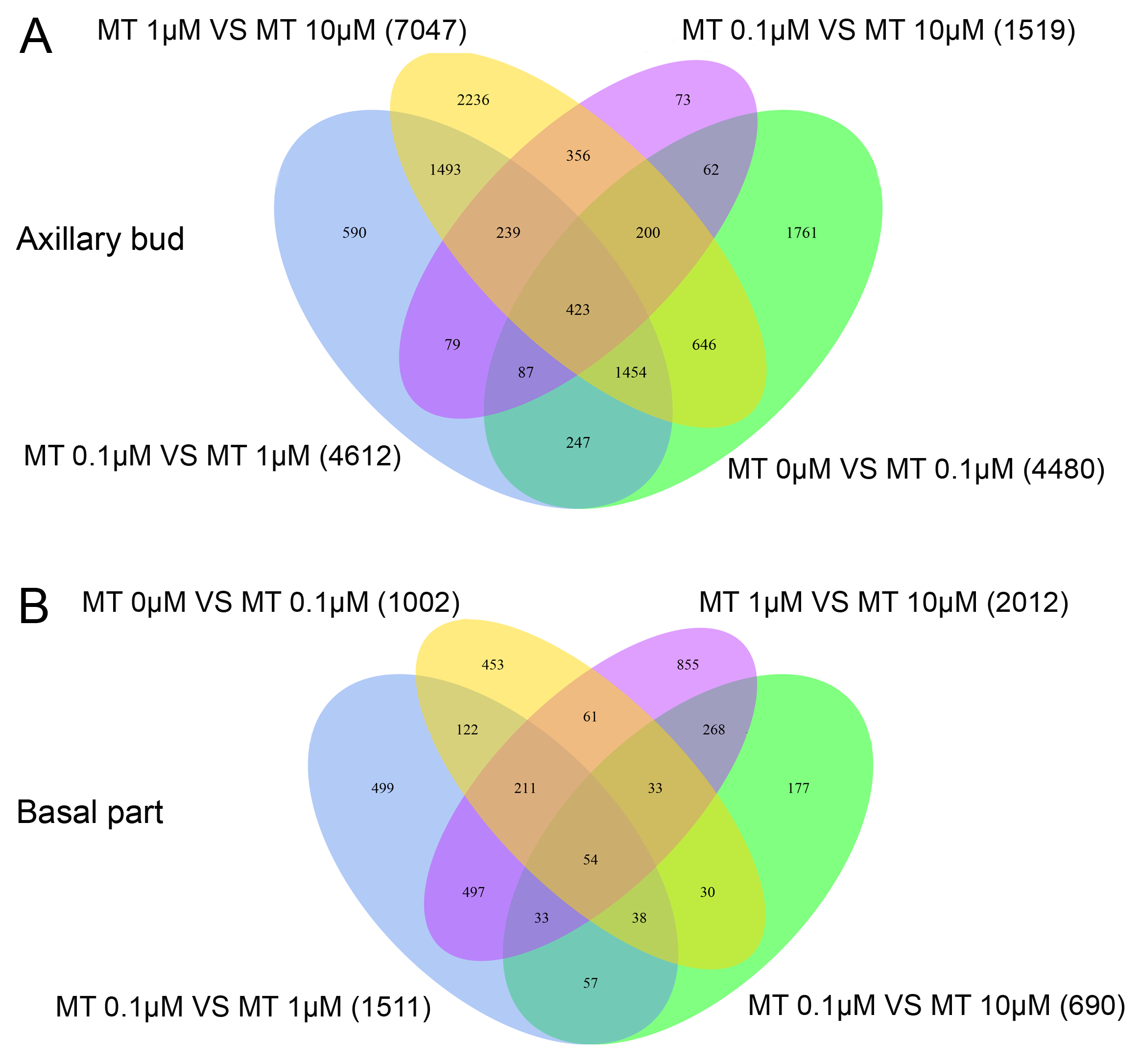
*

**Supplementary Figure 1.** Venn diagrams of differential expression genes in the axillary bud (A) and basal part (B) under different concentrations of melatonin. MT 0 μM VS MT 0.1 μM represents 0 μM melatonin compared to 0.1 μM melatonin. MT 0.1 μM VS MT 1 μM represents 0.1 μM melatonin compared to 1 μM melatonin. MT 0.1 μM VS MT 10 μM represents 0.1 μM melatonin compared to 10 μM melatonin. MT 1 μM VS MT 10 μM represents 1 μM melatonin compared to 10 μM melatonin. MT represents melatonin.

**
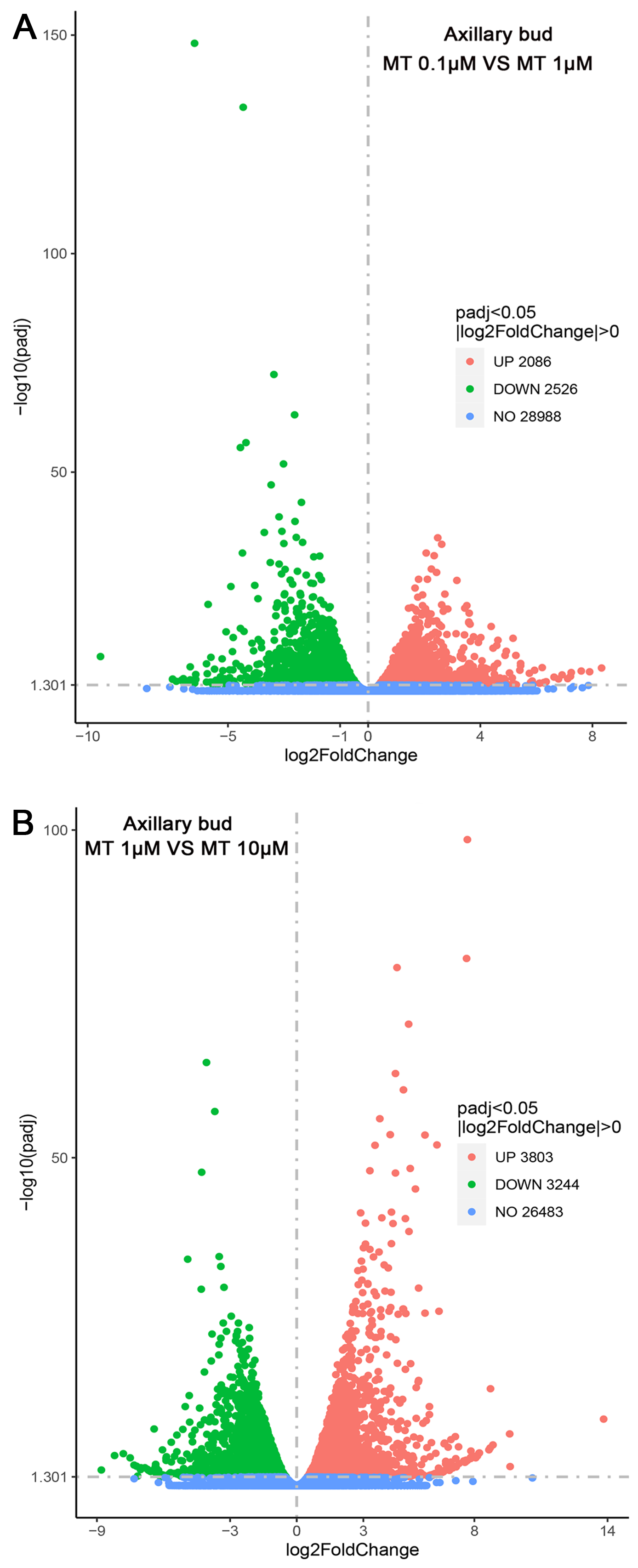
**

**Supplementary Figure 2.** Volcano plot of differentially expressed genes (DEGs) in axillary bud under different concentrations of melatonin. DEGs at 0.1 μM melatonin compared to 1 μM melatonin (A), and 1 μM melatonin compared to 10 μM melatonin (B), The abscissa represents the change of gene expression and multiple (log_2_ Fold Change), and the ordinate represents the significant level of DEGs (−log_10_ False Discovery Rate). Red color in figure indicates up-regulated genes, green color in figure indicates down-regulated genes, and blue color in figure indicates no differentially expressed genes. MT represents melatonin.


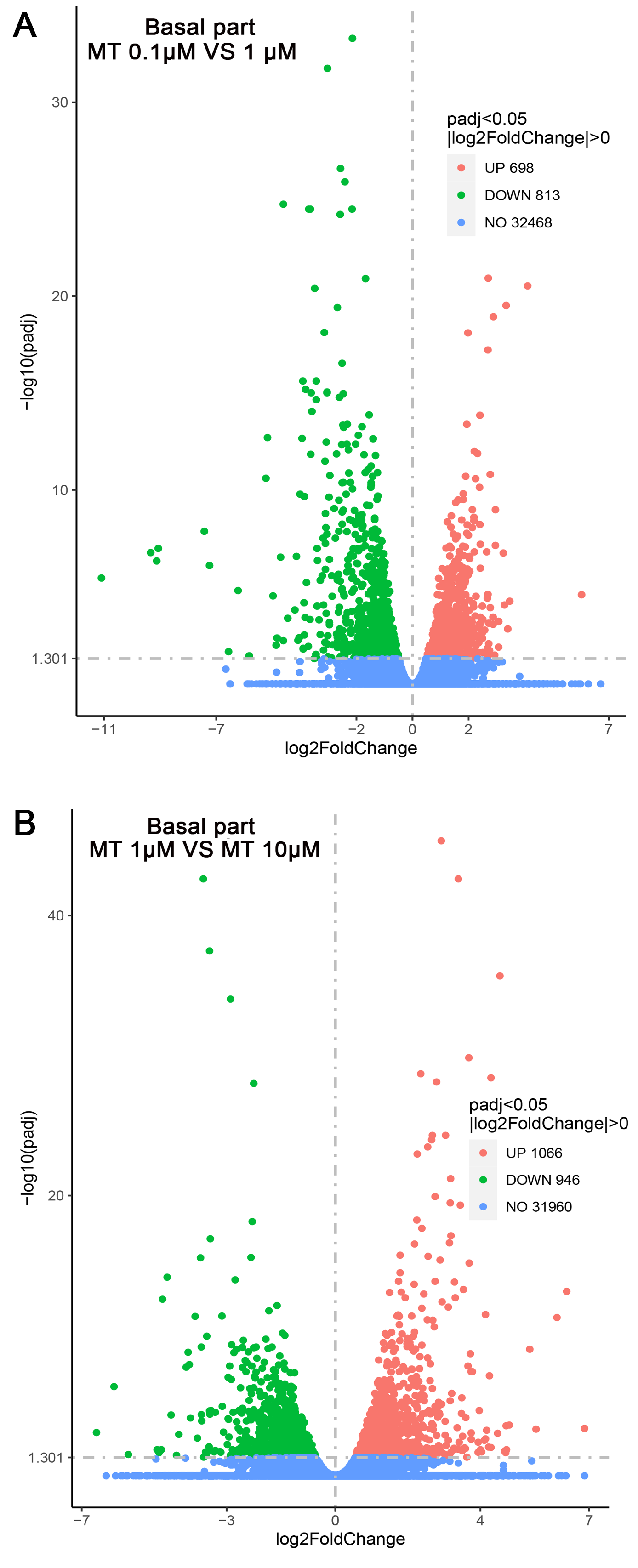


**Supplementary Figure 3.** Volcano plot of differentially expressed genes (DEGs) in basal part under different concentrations of melatonin. DEGs at 0.1 μM melatonin compared to 1 μM melatonin (A), and 1 μM melatonin compare to 10 μM melatonin (B). The abscissa represents the change of gene expression and multiple (log_2_ Fold Change), and the ordinate represents the significant level of DEGs (−log_10_ False Discovery Rate). Red color in figure indicates up-regulated genes, green color in figure indicates down-regulated genes, and blue color in figure indicates no differentially expressed genes. MT represents melatonin.


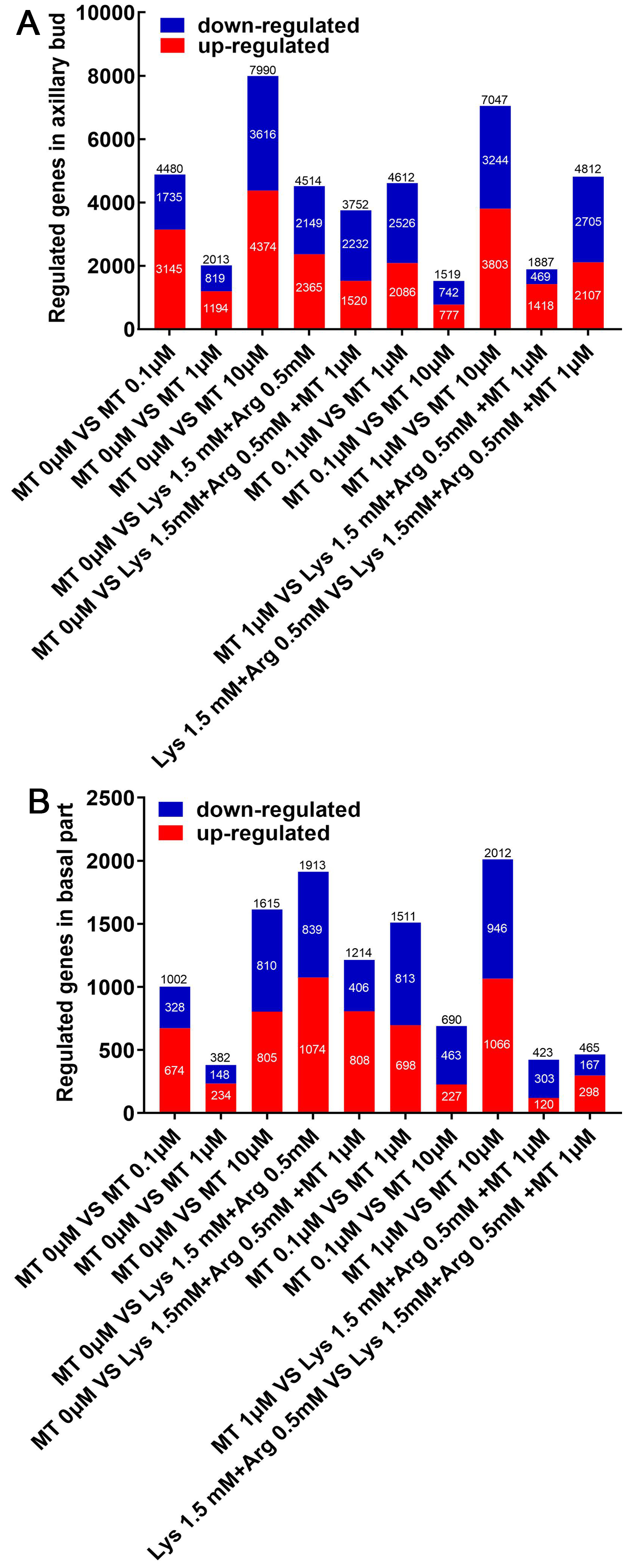


**Supplementary Figure 4.** Differentially expressed genes (DEGs) in axillary bud and basal part under different comparisons. The number of up and down-regulated DEGs (fold change> 2 and padj< 0.05 by DESeq2) detected under different concentrations of melatonin comparisons (0, 0.1, 1, 10 μM) and different concentrations of amino acids related treatments comparisons ( 1.5 mM Lys + 0.5 mM Arg, or 1.5 mM Lys + 0.5 mM Arg + 1 μM melatonin) in the axillary bud (A) and basal part (B). Blue dots represent down-regulated genes, and red dots represent up-regulated genes. MT represents melatonin, Lys represents lysine, and Arg represents arginine.


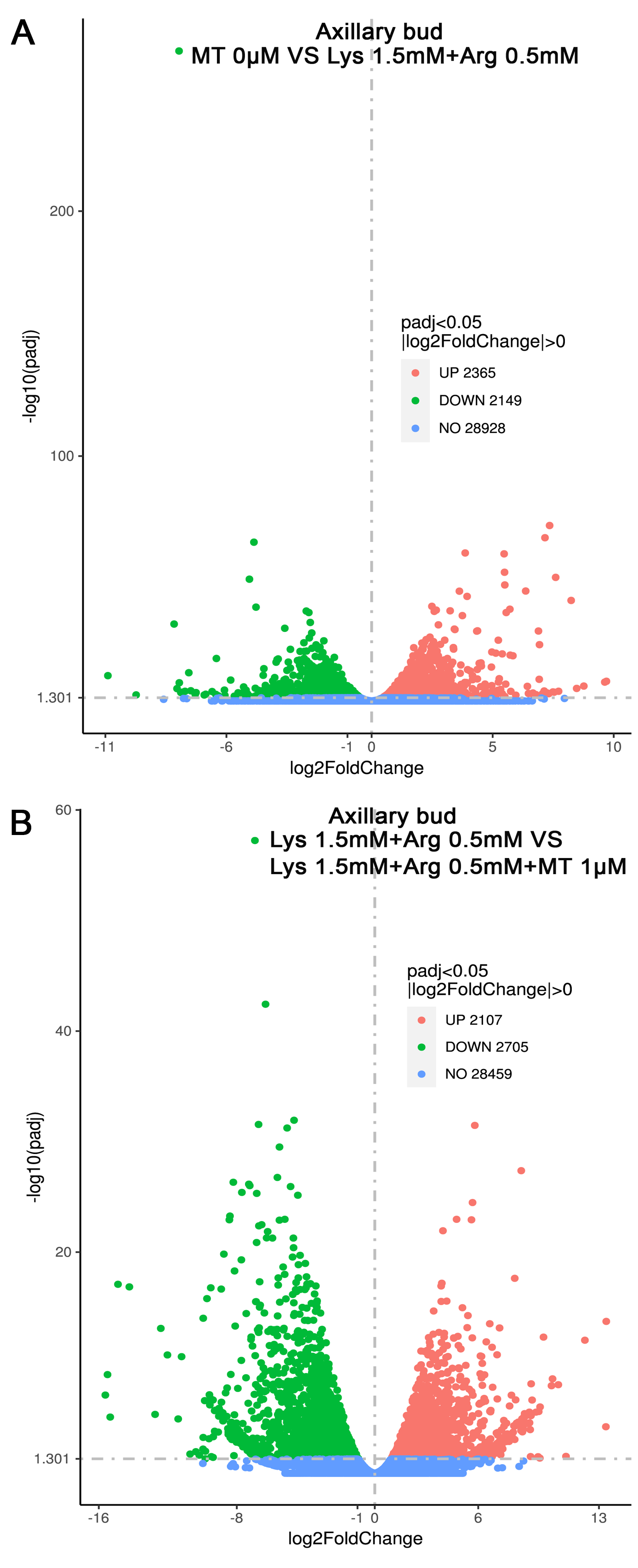


**Supplementary Figure 5.** Volcano plot of differential expression genes (DEGs) in axillary bud under amino acids related treatments. DEGs at 0 μM melatonin compared to 1.5 mM Lys + 0.5 mM Arg (A), and 1.5 mM Lys + 0.5 mM Arg compared to 1.5 mM Lys + 0.5 mM Arg + 1 μM melatonin (B). The abscissa represents the change of gene expression and multiple (log_2_ Fold Change), and the ordinate represents the significant level of DEGs (−log_10_ False Discovery Rate). Red color in figure indicates up-regulated genes, green color in figure indicates down-regulated genes, and blue color in figure indicates no differentially expressed genes. MT represents melatonin, Lys represents lysine, and Arg represents arginine.


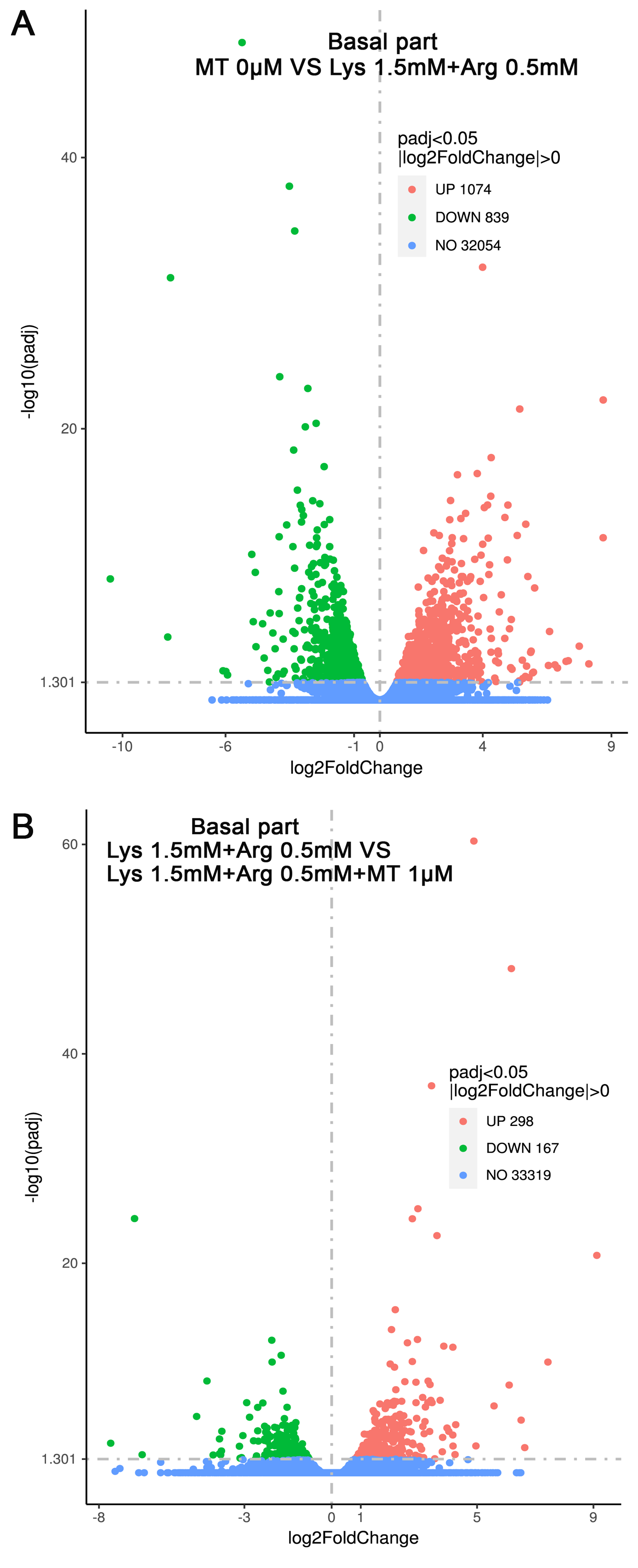


**Supplementary Figure 6.** Volcano plot of differential expression genes (DEGs) in basal part under amino acids related treatments. DEGs at 0 μM melatonin compared to 1.5 mM Lys + 0.5 mM Arg (A), and 1.5 mM Lys + 0.5 mM Arg compared to 1.5 mM Lys + 0.5 mM Arg + 1 μM melatonin (B). The abscissa represents the change of gene expression and multiple (log_2_ Fold Change), and the ordinate represents the significant level of DEGs (−log_10_ False Discovery Rate). Red color in figure indicates up-regulated genes, green color in figure indicates down-regulated genes, and blue color in figure indicates no differentially expressed genes. MT represents melatonin, Lys represents lysine, and Arg represents arginine.


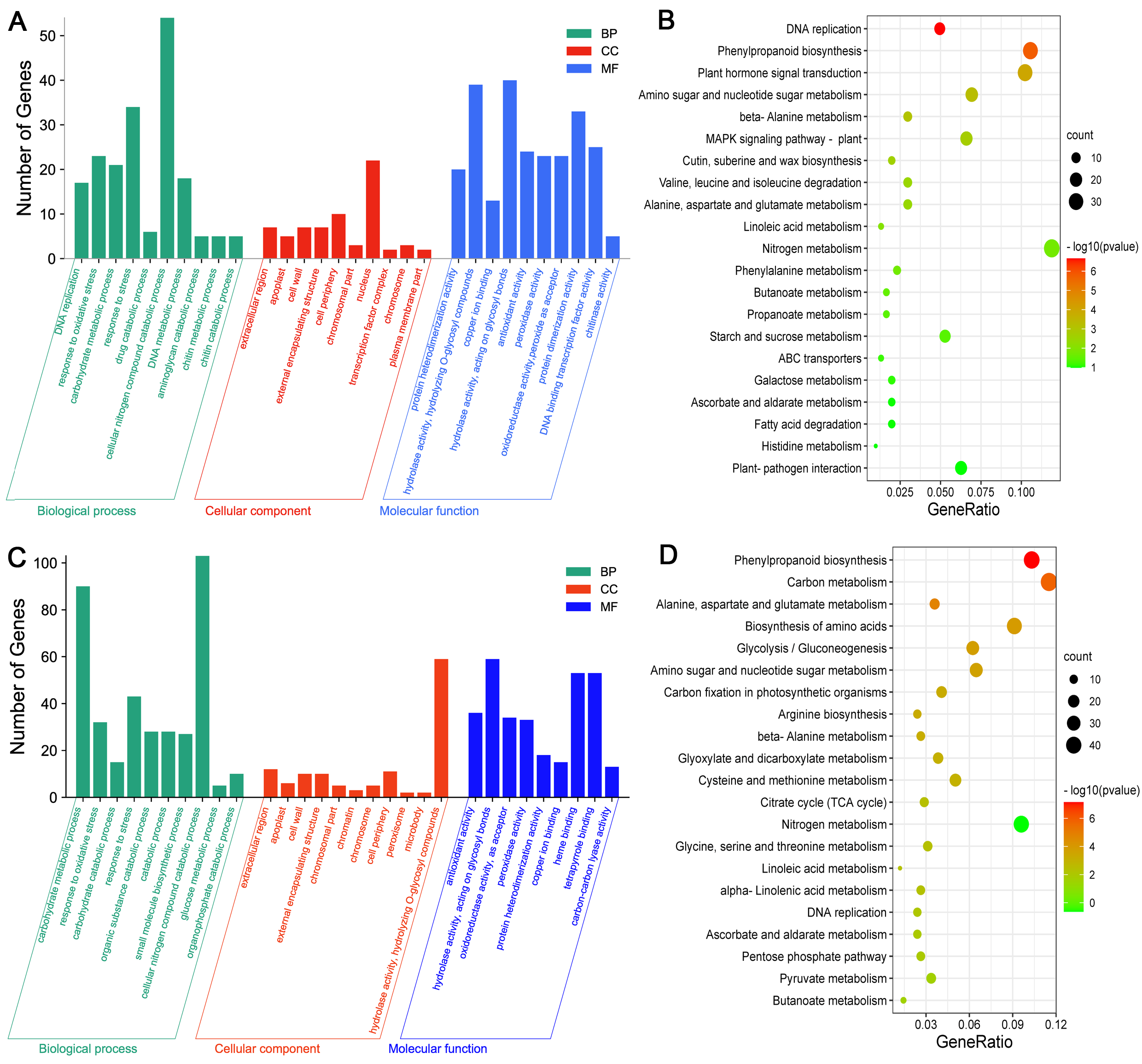


**Supplementary Figure 7.** Analysis of differential expression genes (DEGs) with different expression patterns in basal part under different concentrations of melatonin. The GO enrichment (A) and KEGG pathway enrichment (B) analysis of DEGs at 0.1 μM melatonin compared to 1 μM melatonin. The GO enrichment (C) and KEGG pathway enrichment (D) analysis of DEGs at 1 μM melatonin compared to 10 μM melatonin. Y-axis indicates the number of GO annotated genes, and X-axis indicates the processes/components in different biological processes, cellular components, and molecular functions (A, C). Y-axis indicates KEGG pathway and X-axis indicates the ratio of the number of enriched genes to the number of annotated genes in the pathway (B, D). The color of the dot represents p-value, and the size of the dot represents the number of DEGs mapped to the referent pathway.


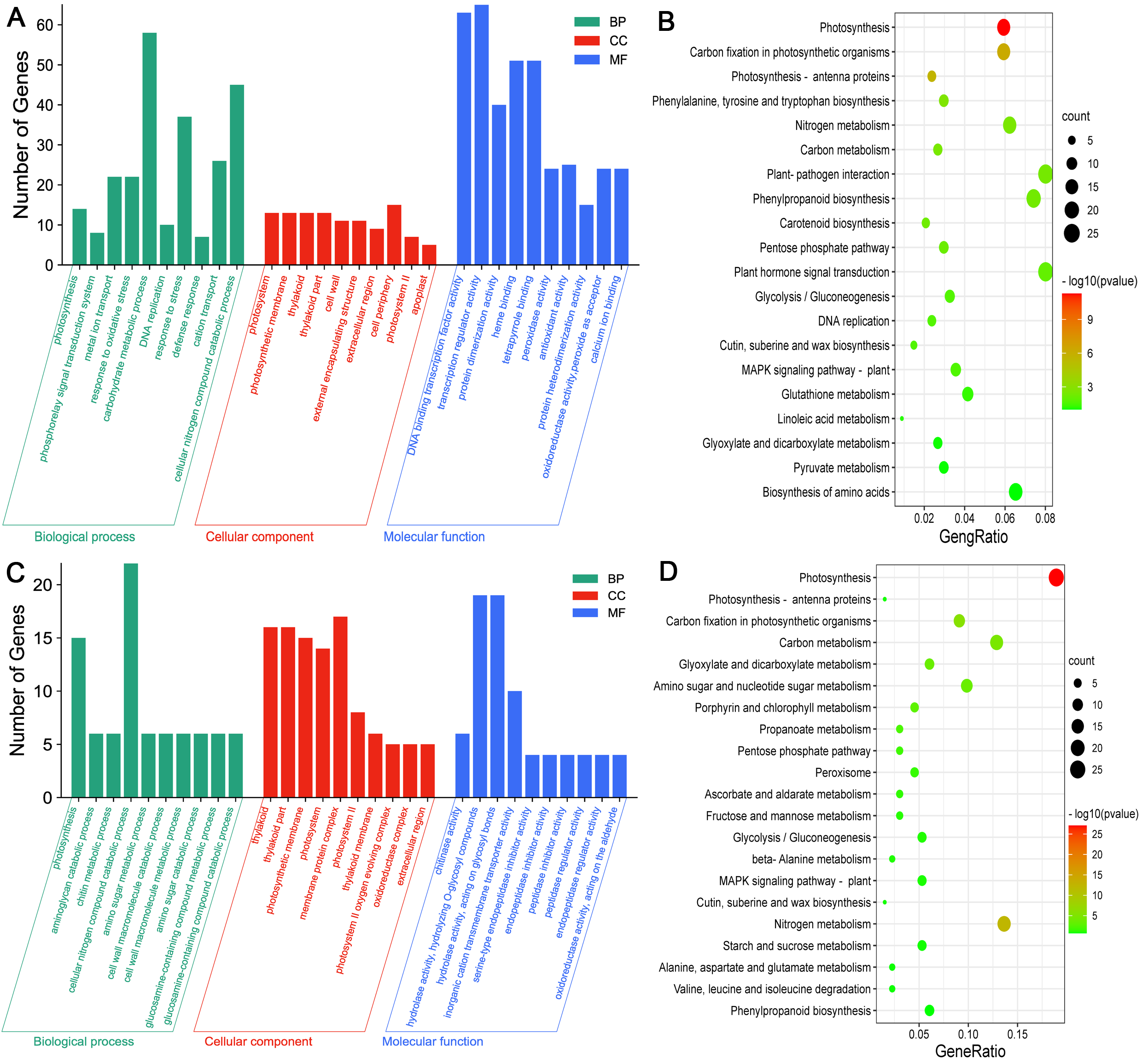


**Supplementary Figure 8.** Analysis of differential expression genes (DEGs) with different expression patterns in basal part under amino acids related treatments. The GO enrichment (A) and KEGG pathway enrichment (B) analysis of DEGs at 0 μM melatonin compared to 1.5 mM Lys + 0.5 mM Arg. The GO enrichment (C) and KEGG pathway enrichment (D) analysis of DEGs at 1.5 mM Lys + 0.5 mM Arg compared to 1.5 mM Lys + 0.5 mM Arg + 1 μM melatonin. Y-axis indicates the number of GO annotated genes, and X-axis indicates the processes/components in different biological processes, cellular components, and molecular functions (A, C). Y-axis indicates KEGG pathway, and X-axis indicates the ratio of the number of enriched genes to the number of annotated genes in the pathway (B, D). The color of the dot represents p-value, and the size of the dot represents the number of DEGs mapped to the referent pathway.


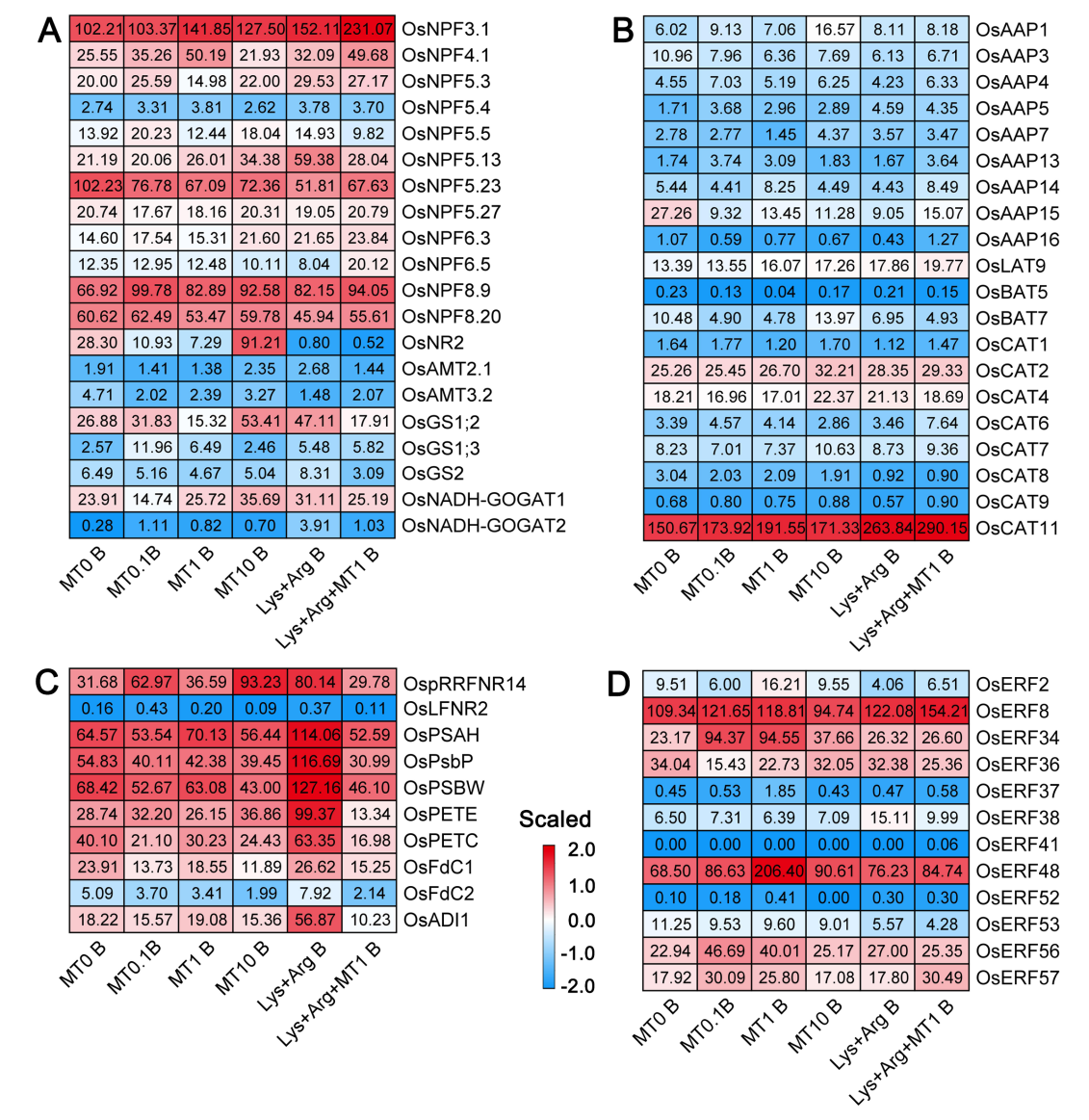


**Supplementary Figure 9.** The expression of genes involved in nitrogen metabolism, photosynthesis and stress in basal part under different concentrations of melatonin and amino acids. Heatmaps display the expression patterns of genes involved in nitrate and ammonium transport and assimilation (A), amino acid transport (B), photosynthesis (C) and stress (D). Red and blue represent the highest and lowest level of expression. MT represents melatonin, Lys represents lysine, and Arg represents arginine. A at the bottom of each graph represents axillary bud, and B at the bottom of each graph represents basal part. MT0, MT0.1, MT1 and MT10 represents melatonin at 0 μM, 0.1 μM, 1 μM and 10 μM respectively.


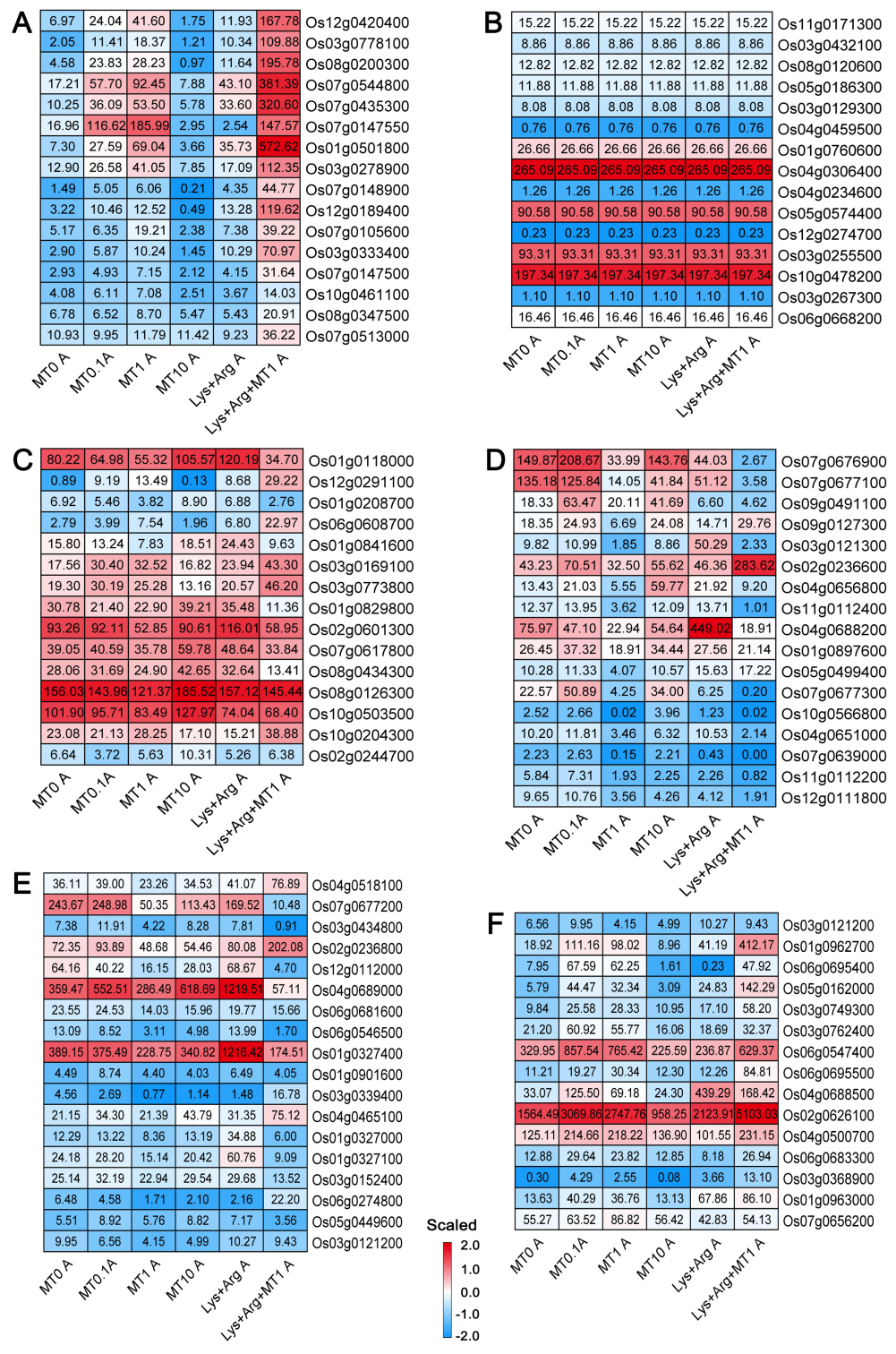


**Supplementary Figure 10.** The expression of genes involved in photosynthesis, carbon metabolism, and phenylpropanoid biosynthesis in axillary bud under different concentrations of melatonin and amino acids. Heatmaps display the expression patterns of genes involved in photosynthesis (A), carbon metabolism (B, C), and phenylpropanoid biosynthesis (D, E, F). Red and blue represent the highest and lowest level of expression. MT represents melatonin, Lys represents lysine, and Arg represents arginine. A at the bottom of each graph represents axillary bud, and B at the bottom of each graph represents basal part. MT0, MT0.1, MT1 and MT10 represents melatonin at 0 μM, 0.1 μM, 1 μM and 10 μM respectively.


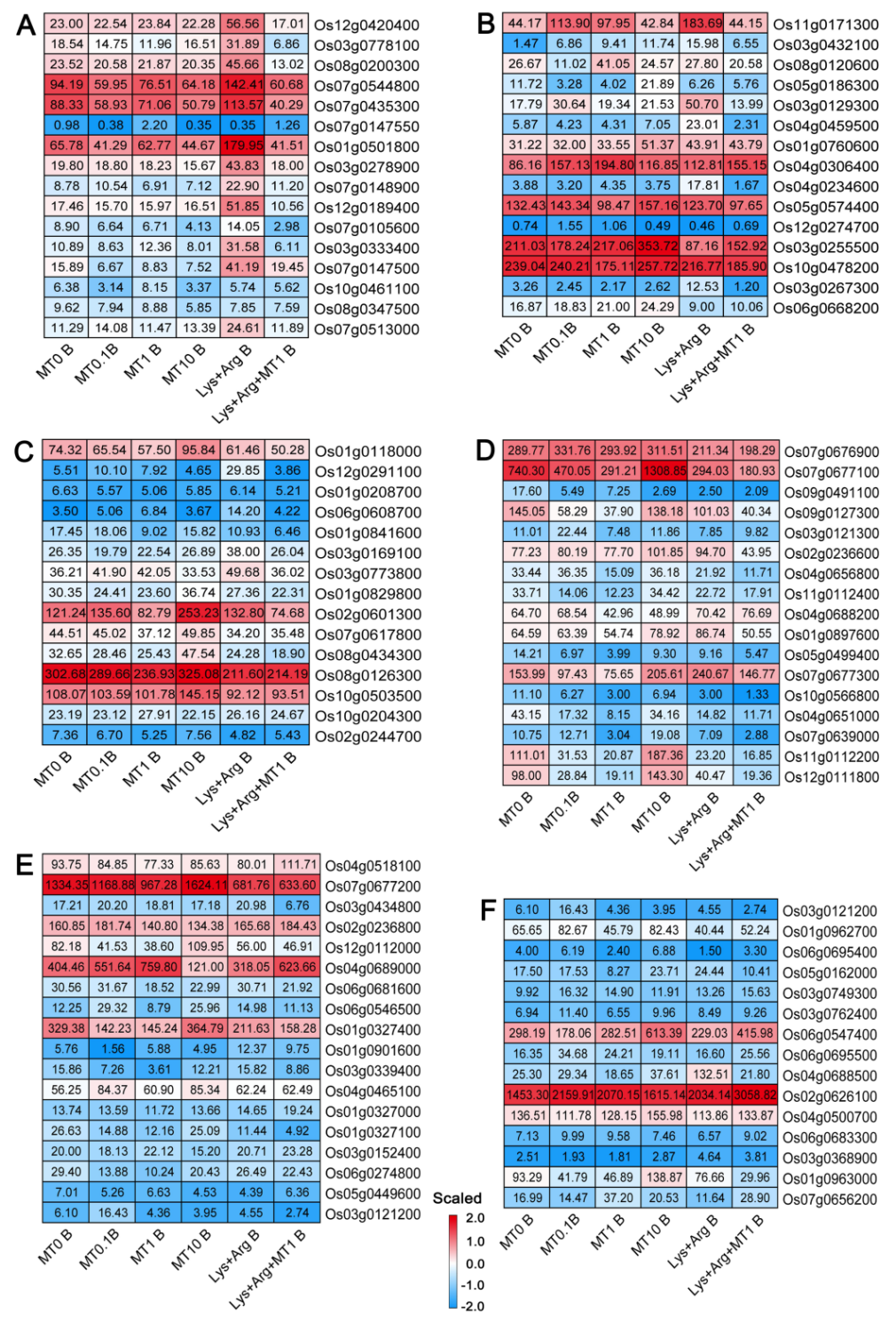


**Supplementary Figure 11.** The expression of genes involved in photosynthesis, carbon metabolism, and phenylpropanoid biosynthesis in basal part under different concentrations of melatonin and amino acids. Heatmaps display the expression patterns of genes involved in photosynthesis (A), carbon metabolism (B, C), and phenylpropanoid biosynthesis (D, E, F). Red and blue represent the highest and lowest level of expression. MT represents melatonin, Lys represents lysine, and Arg represents arginine. A at the bottom of each graph represents axillary bud, and B at the bottom of each graph represents basal part. MT0, MT0.1, MT1 and MT10 represents melatonin at 0 μM, 0.1 μM, 1 μM and 10 μM respectively.


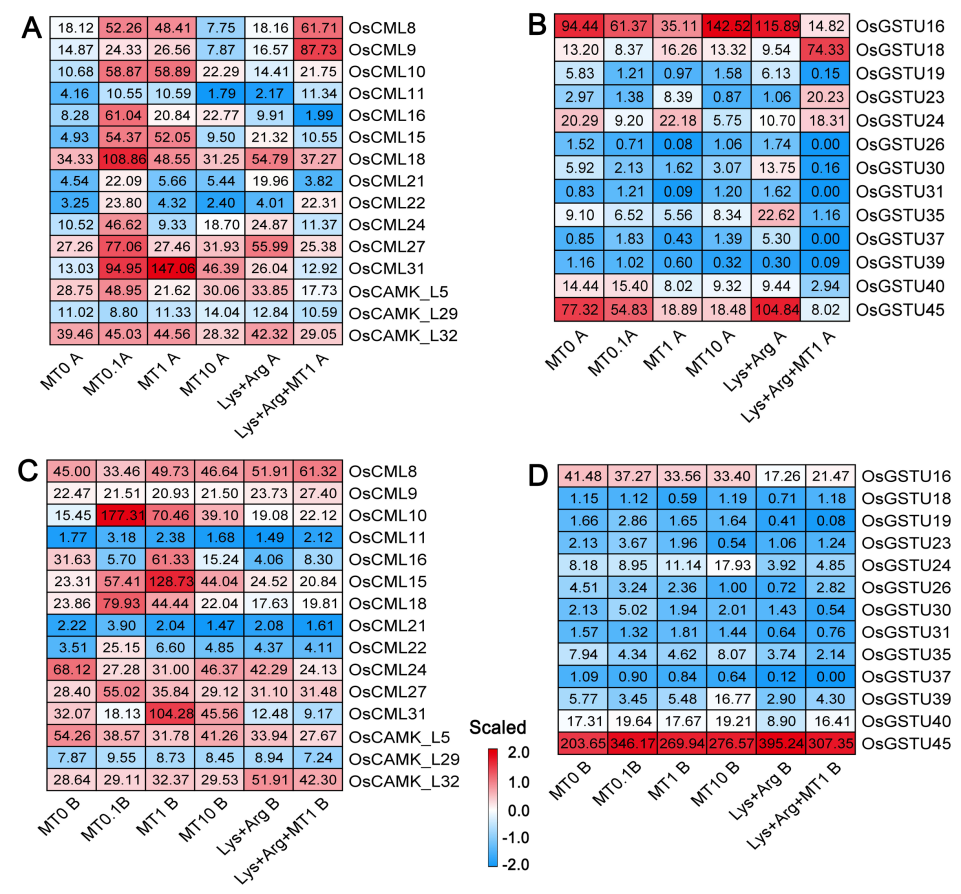


**Supplementary Figure 12.** The expression of genes involved in calmodulin and glutathione S-transferase in axillary bud and basal part under different concentrations of melatonin and amino acids. Heatmaps display the expression patterns of genes involved in calmodulin (A, C), glutathione S-transferase (B, D) in axillary bud (A, B) and basal part (C, D). Red and blue represent the highest and lowest level of expression. MT represents melatonin, Lys represents lysine, and Arg represents arginine. A at the bottom of each graph represents axillary bud, and B at the bottom of each graph represents basal part. MT0, MT0.1, MT1 and MT10 represents melatonin at 0 μM, 0.1 μM, 1 μM and 10 μM respectively.


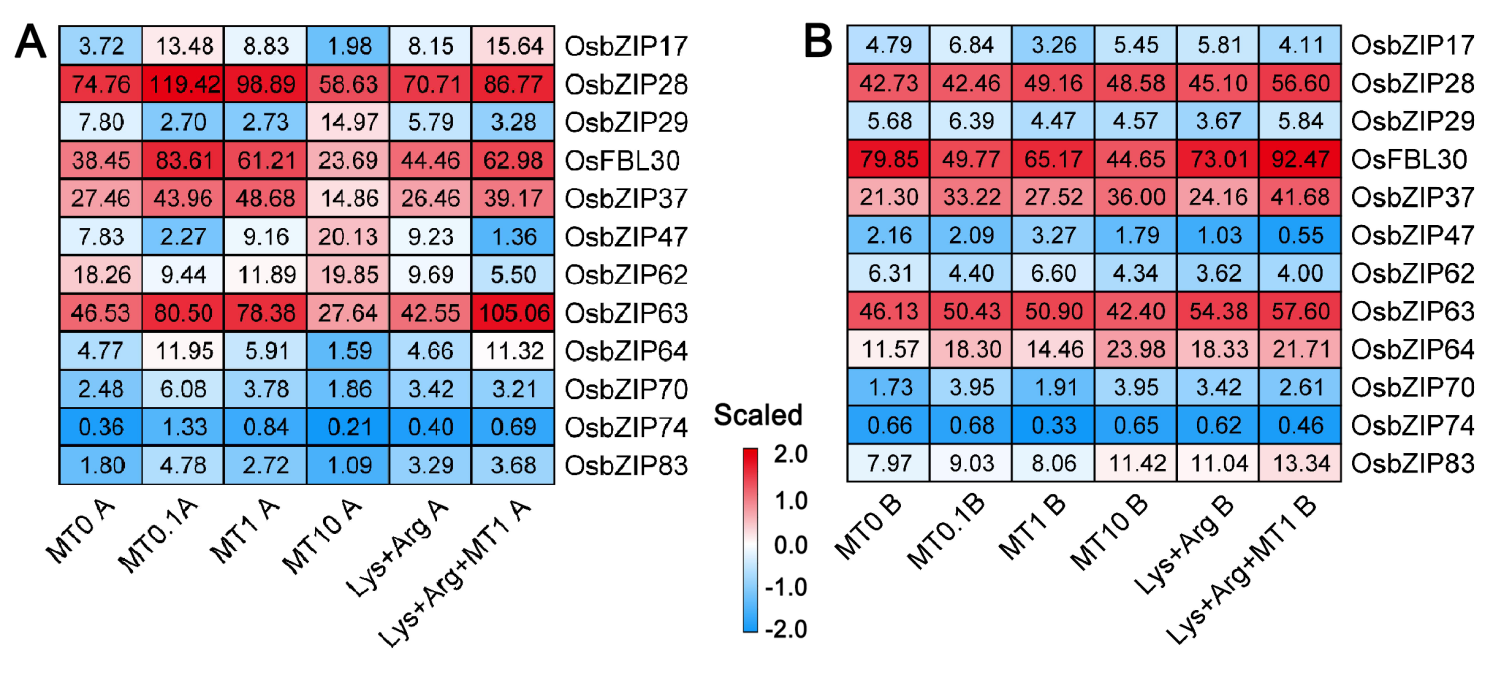


**Supplementary Figure 13.** The expression of genes involved in plant hormone signal transduction in axillary bud and basal part under different concentrations of melatonin and amino acids. Heatmaps display the expression patterns of genes involved in axillary bud (A) and basal part (B) of basic leucine zipper transcription factor in abscisic acid pathway. Red and blue represent the highest and lowest level of expression. MT represents melatonin, Lys represents lysine, and Arg represents arginine. A at the bottom of each graph represents axillary bud, and B at the bottom of each graph represents basal part. MT0, MT0.1, MT1 and MT10 represents melatonin at 0 μM, 0.1 μM, 1 μM and 10 μM respectively.


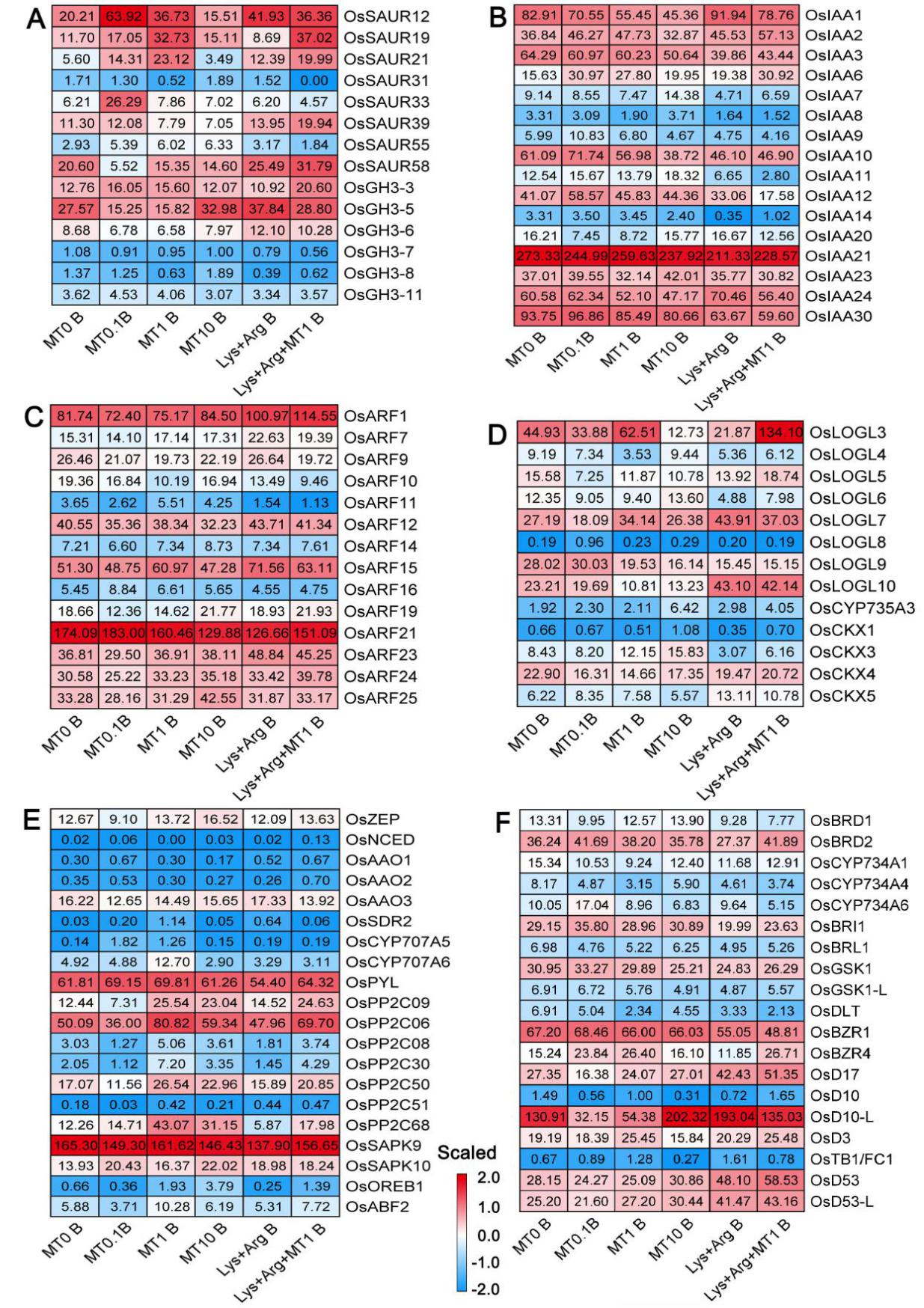


**Supplementary Figure 14.** The expression of genes involved in plant hormone signal transduction in basal part under different concentrations of melatonin and amino acids. Heatmaps display the expression patterns of genes involved in auxin (A-C), cytokinin (D), abscisic acid (E), brassinosteroids and strigolactone (F). Red and blue represent the highest and lowest level of expression. MT represents melatonin, Lys represents lysine, and Arg represents arginine. A at the bottom of each graph represents axillary bud, and B at the bottom of each graph represents basal part. MT represents melatonin, Lys represents lysine, and Arg represents arginine. A at the bottom of each graph represents axillary bud, and B at the bottom of each graph represents basal part. MT0, MT0.1, MT1 and MT10 represents melatonin at 0 μM, 0.1 μM, 1 μM and 10 μM respectively.


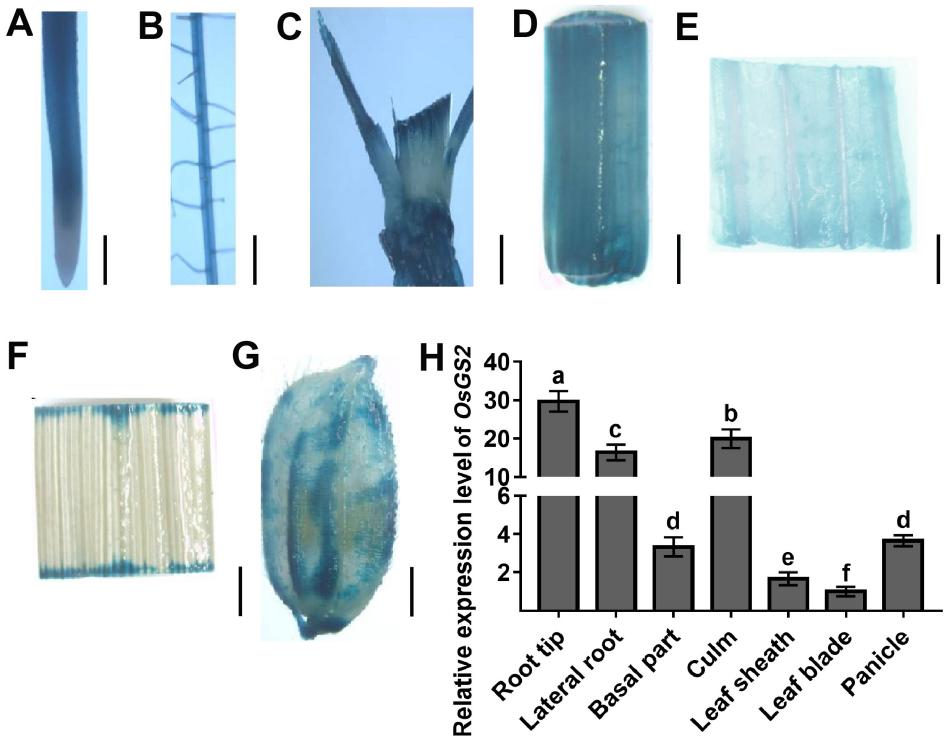


**Supplementary Figure 15.** The expression analysis of *OsGS2* in rice promoter-GUS and wild-type ZH11. GUS staining under the control of the *OsGS2* promoter was detected in root tip (A), lateral root (B), basal part (C), culm (D), leaf sheath (E), leaf blade (F) and panicle (G). And the expression analysis of *OsGS2* was detected by RT-qPCR in wild-type ZH11 (H). Values are means ± SD (n=4), and the significance levels of different lowercase letters were as follows, *P* < 0.05. Bars = 1 mm.


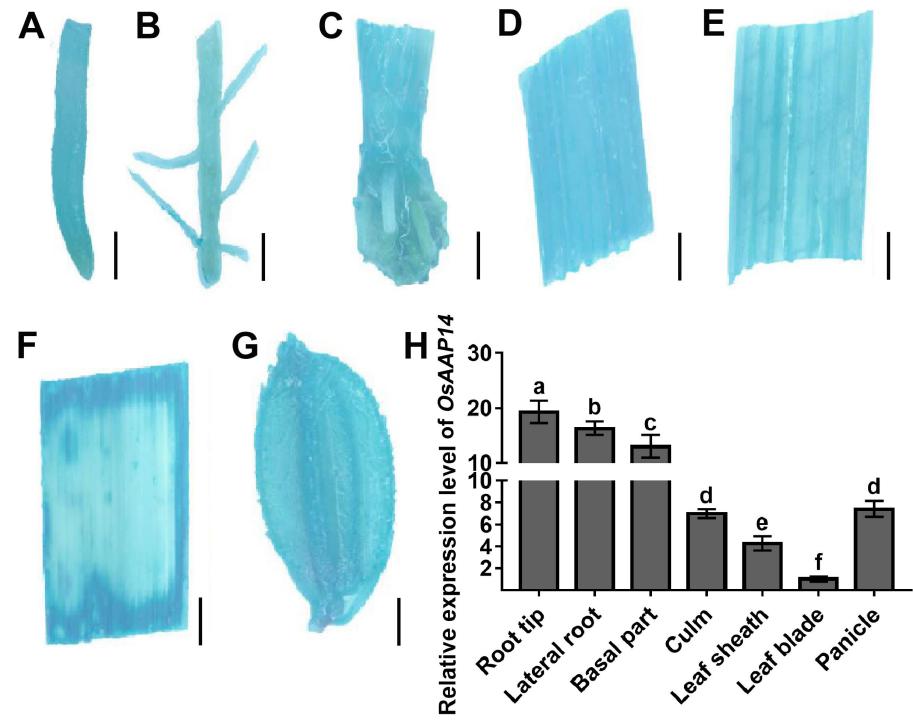


**Supplementary Figure 16.** The expression analysis of *OsAAP14* in rice promoter-GUS and wild-type ZH11. GUS staining under the control of the *OsAAP14* promoter was detected in root tip (A), lateral root (B), basal part (C), culm (D), leaf sheath (E), leaf blade (F) and panicle (G). And the expression analysis of *OsAAP14* was detected by RT-qPCR in wild-type ZH11 (H). Values are means ± SD (n=4), and the significance levels of different lowercase letters were as follows, *P* < 0.05. Bars = 1 mm.


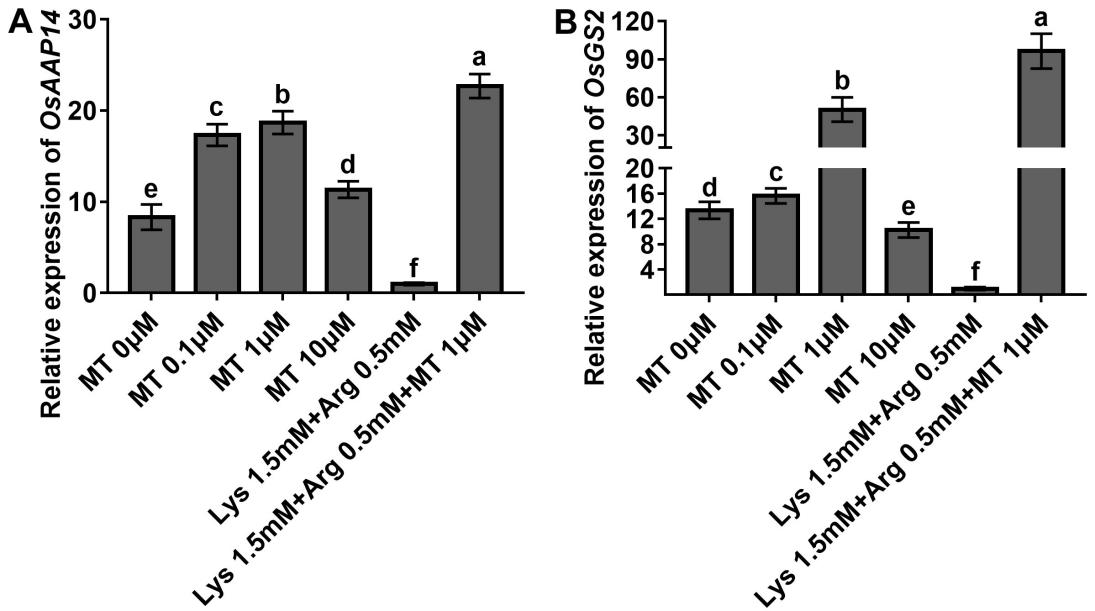


**Supplementary Figure 17.** Relative expression of *OsAAP14* and *OsGS2* in the rice root is regulated by melatonin and amino acids. Relative expression of *OsAAP14* (A) and *OsGS2* (B) in the rice root was treated with basic nutrient solutions supplemented with different concentrations of melatonin (0 μM, 0.1 μM, 1 μM, 10 μM), 1.5 mM Lys + 0.5 mM Arg, or 1.5 mM Lys + 0.5 mM Arg + 1 μM melatonin for 2 h, respectively. Values are means ± SD (n=4), and the significance levels of different lowercase letters were as follows, *P* < 0.05. MT represents melatonin, Lys represents lysine, and Arg represents arginine.


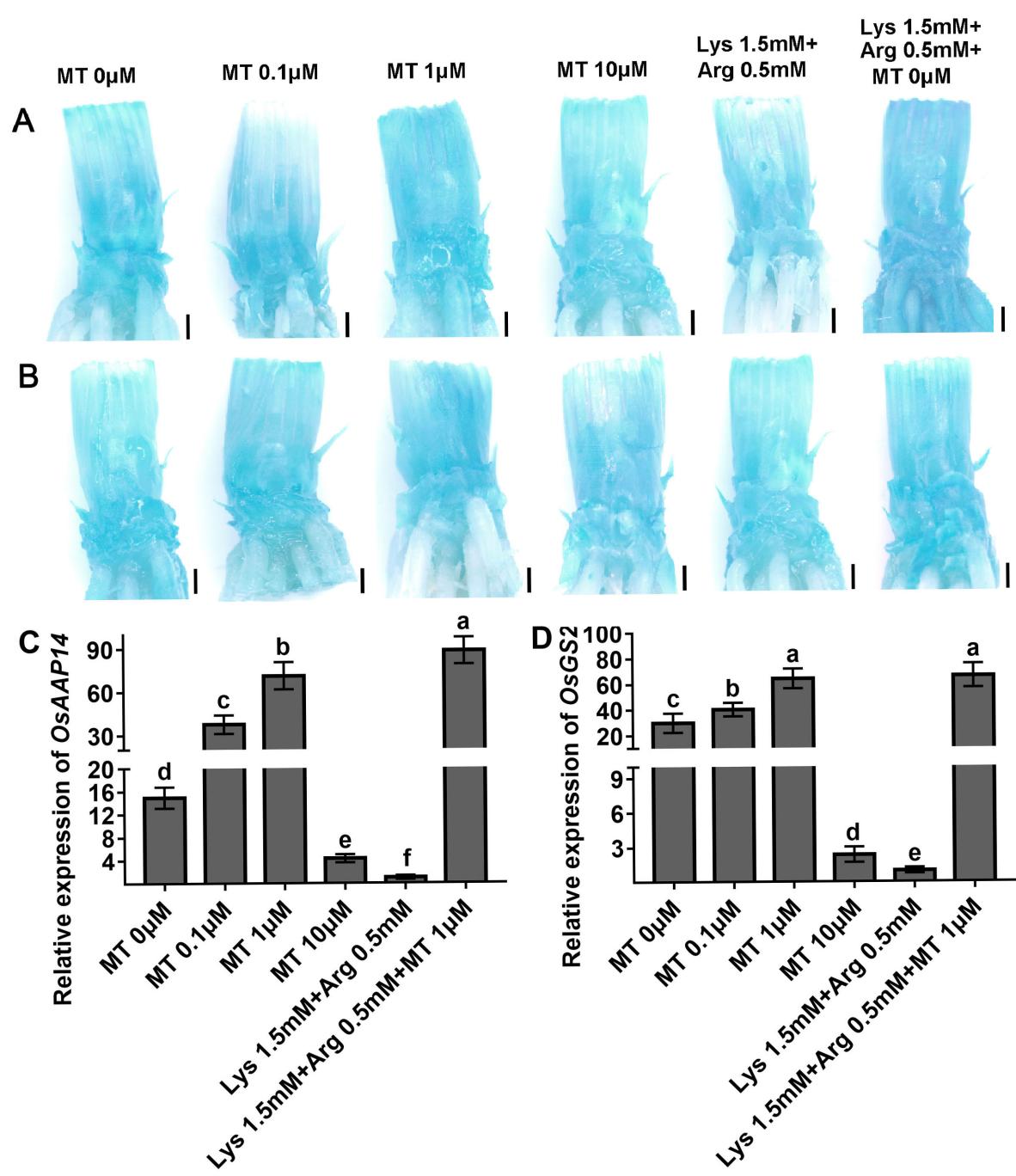


**Supplementary Figure 18.** The expression of *OsAAP14* and *OsGS2* in the basal part and axillary bud is regulated by melatonin and amino acids. *OsAAP14* (A) and *OsGS2* (B) promoter-GUS staining in the basal part and axillary bud were treated with basic nutrient solutions supplemented with different concentrations of melatonin (0 μM, 0.1 μM, 1 μM, 10 μM), 1.5 mM Lys + 0.5 mM Arg, or 1.5 mM Lys + 0.5 mM Arg + 1 μM melatonin for 2 h, respectively. Relative expression of *OsAAP14* (C) and *OsGS2* (D) in the basal part and axillary bud were treated with basic nutrient solutions supplemented with different concentrations of melatonin (0 μM, 0.1 μM, 1 μM, 10 μM), 1.5 mM Lys + 0.5 mM Arg, or 1.5 mM Lys + 0.5 mM Arg + 1 μM melatonin for 2 h, respectively. Values are means ± SD (n=4), and the significance levels of different lowercase letters were as follows, *P* < 0.05. MT represents melatonin, Lys represents lysine, and Arg represents arginine. Bars = 1 mm.

**
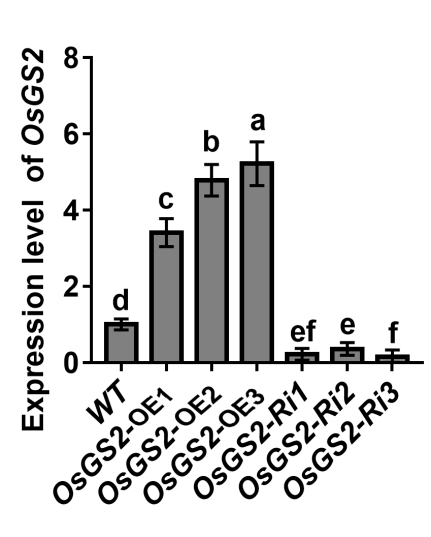
**

**Supplementary Figure 19.** The expression level of over-expression and Ri lines of *OsGS2* in ZH11 background. Values are means ± SD (n=4), and the significance levels of different lowercase letters were as follows, *P* < 0.05. WT represents wild-type ZH11, *OsGS2* OE1-OE3 represent three lines of *OsGS2* over-expressing transgenic plants, and *OsGS2* Ri1-Ri3 represent three lines of *OsGS2* Ri transgenic plants.


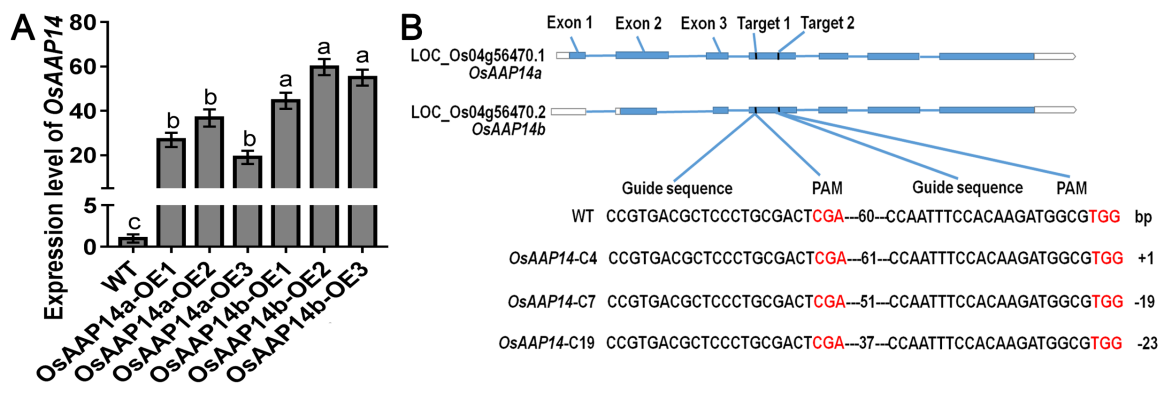


**Supplementary Figure 20.** Identification of over-expression and CRISPR mutant lines of *OsAAP14* in ZH11 background. The expression level of *OsAAP14* in over-expression transgenic plants (A) and sequencing of base deletion and insertion of *OsAAP14* CRISPR mutant lines (B).WT represents wild-type ZH11, *OsAAP14a-*OE1-OE3 represent three lines of *OsAAP14* longer splicing variant over-expressing transgenic plants, *OsAAP14b-*OE1-OE3 represent three lines of *OsAAP14* shorter splicing variant over-expressing transgenic plants, and *OsAAP14-C4*, *OsAAP14-C7* and *OsAAP14-C19* represent three lines of *OsAAP14* CRISPR mutants *osaap14-1*, *osaap14-2* and *osaap14-3*. minus (-) and plus (+) signs indicate the number of nucleotides deleted and inserted at *OsAAP14* CRISPR target sequence site 1 and site 2, respectively. Values are means ± SD (n=4), and the significance levels of different lowercase letters were as follows, *P* < 0.05.


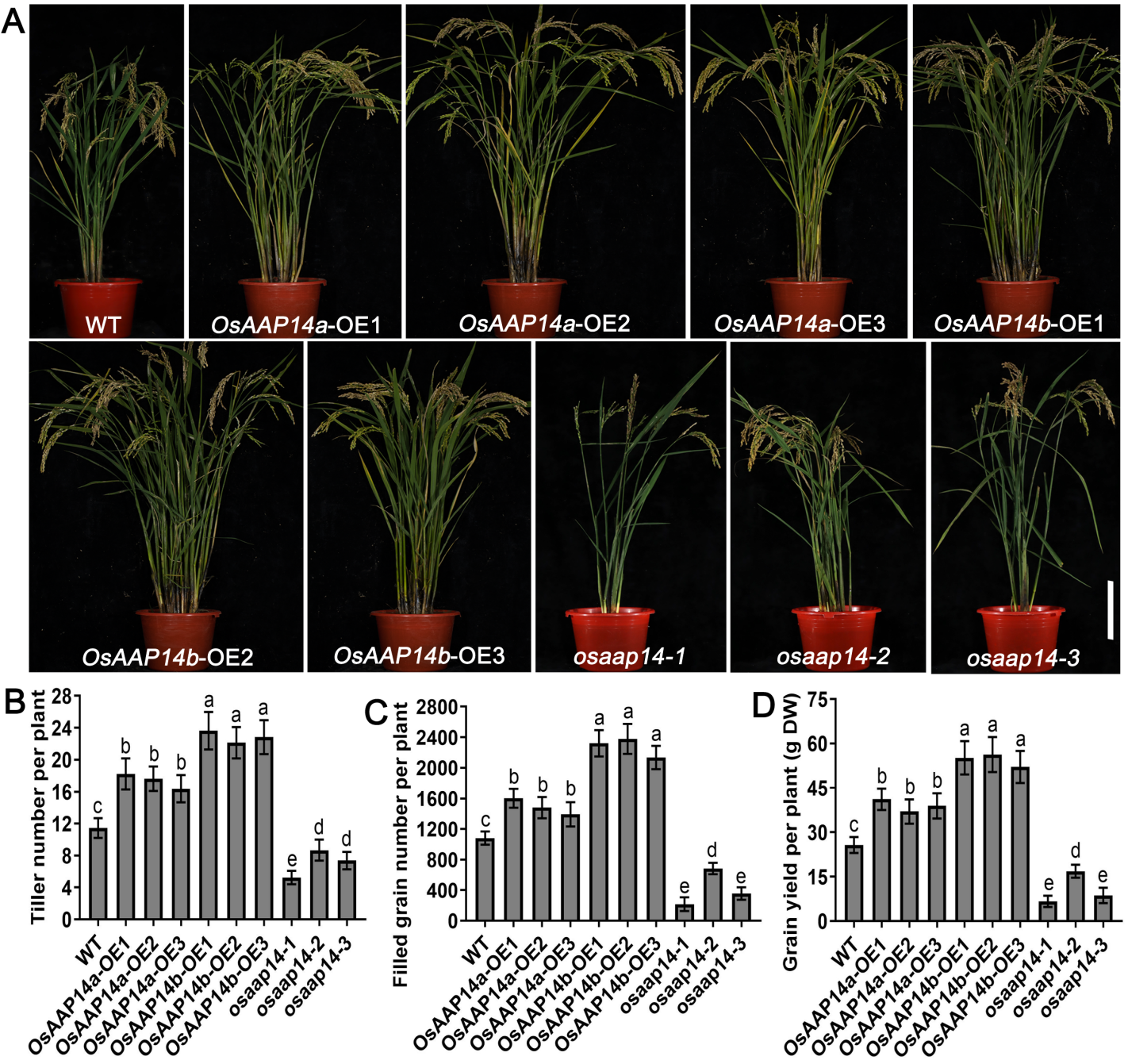


**Supplementary Figure 21.** Phenotypes of *OsAAP14* transgenic plants grown in paddy field. Whole plant phenotypes (A). Statistical analysis of tiller number per plant (B), filled grain number per plant (C) and grain yield per plant (D). WT represents wild-type ZH11, *OsAAP14a-*OE1-OE3 represent three lines of *OsAAP14* longer splicing variant over-expressing transgenic plants, *OsAAP14b-*OE1-OE3 represent three lines of *OsAAP14* shorter splicing variant over-expressing transgenic plants, and *osaap14-1-3* represent three lines of *OsAAP14* CRISPR mutants. Values are means ± SD (n > 20), and the significance levels of different lowercase letters were as follows, *P* < 0.05. Bars = 10 cm.


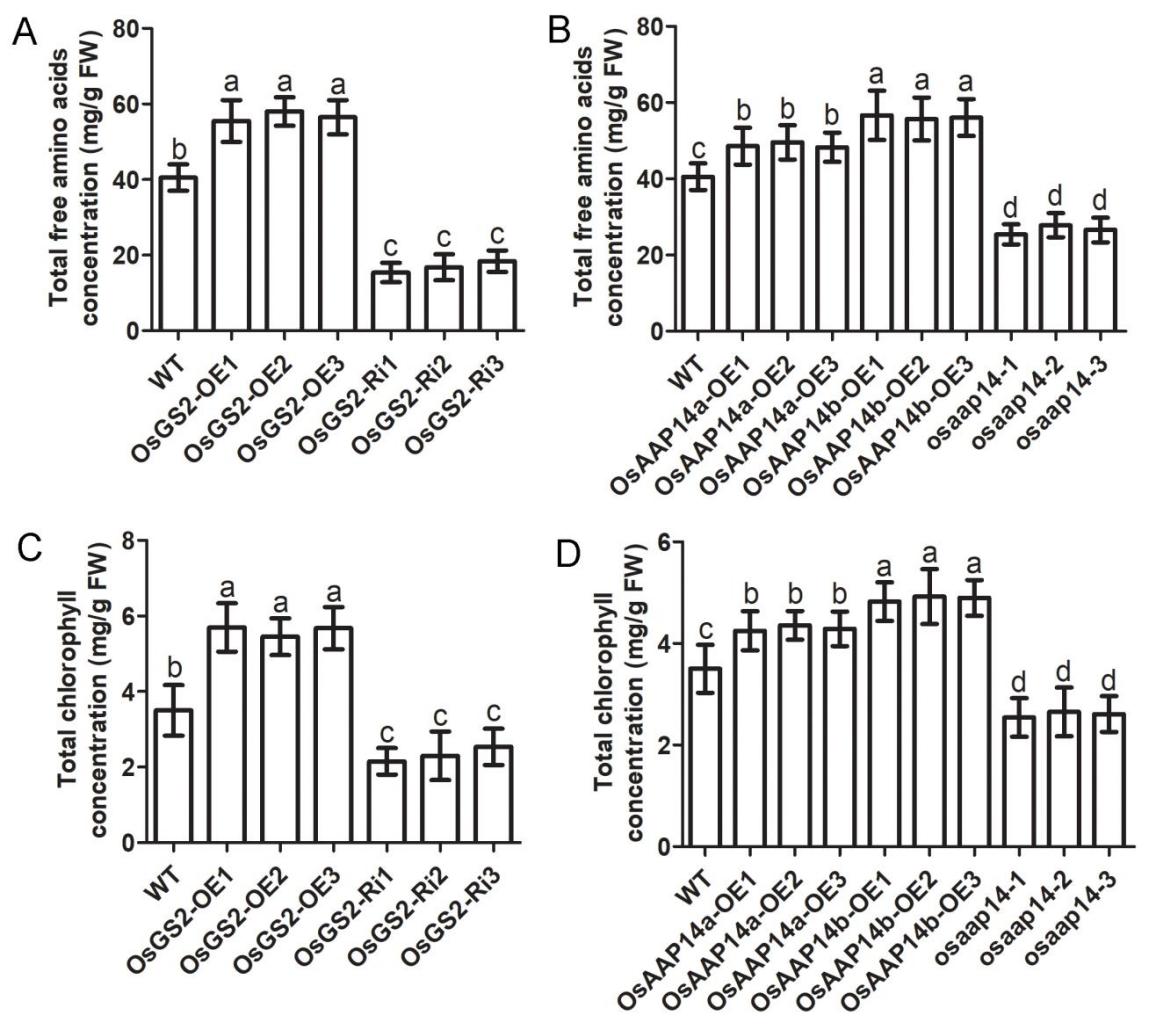


**Supplementary Figure 22.** The analysis of total free amino acids and chlorophyll content in seedlings of *OsGS2* and *OsAAP14* transgenic plants. WT represents wild-type ZH11, *OsGS2* OE1-OE3 represent three lines of *OsGS2* over-expressing transgenic plants, *OsGS2* Ri1-Ri3 represent three lines of *OsGS2* Ri transgenic plants, *OsAAP14a-*OE1-OE3 represent three lines of *OsAAP14* longer splicing variant over-expressing transgenic plants, *OsAAP14b-*OE1-OE3 represent three lines of *OsAAP14* shorter splicing variant over-expressing transgenic plants, and *osaap14-1-3* represent three lines of *OsAAP14* CRISPR mutants after culturing five weeks. Values are means ± SD (n > 6), and the significance levels of different lowercase letters were as follows, *P* < 0.05.


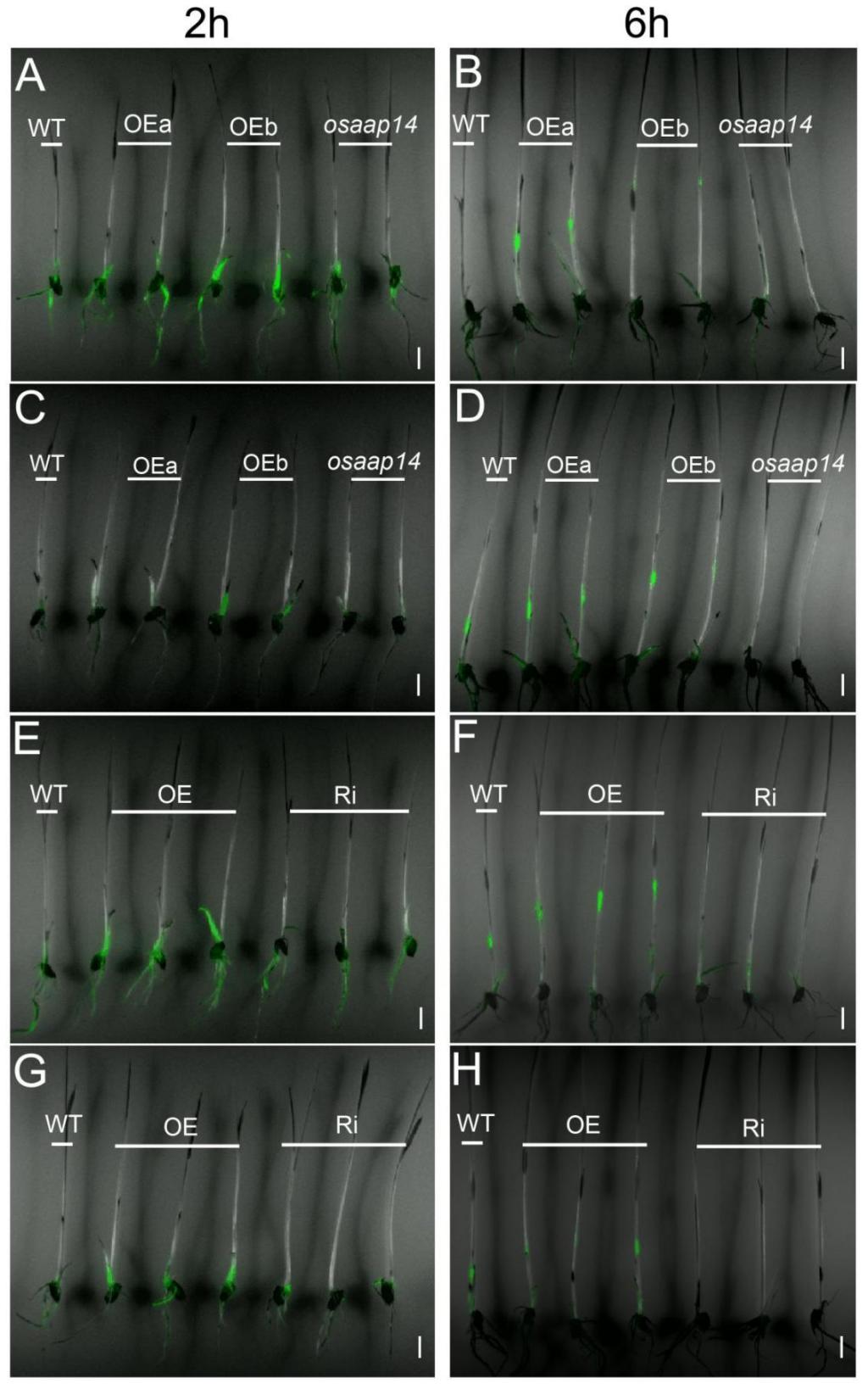


**Supplementary Figure 23.** FITC-labeled amino acid uptake assay in *OsAAP14* and *OsGS2* transgenic plants. Fluorescence was detected after culturing seedlings with FITC-labeled amino acids Arg for 2 h (A) and 6 h (B), and Lys for 2 h (C) and 6 h (D) of *OsAAP14* transgenic plants. Fluorescence was detected after culturing seedlings with FITC-labeled amino acids Arg for 2 h (E) and 6 h (F), and Lys for 2 h (G) and 6 h (H) of *OsGS2* transgenic plants. WT represents wild-type ZH11, OEa represents lines of *OsAAP14* longer splicing variant over-expressing transgenic plants, OEb represents lines of *OsAAP14* shorter splicing variant over-expressing transgenic plants, *osaap14* represents lines of *OsAAP14* CRISPR mutants, OE represents lines of *OsGS2* over-expressing transgenic plants, and Ri represents lines of *OsGS2* Ri transgenic plants. Lys represents lysine, and Arg represents arginine. Bars = 1 cm.


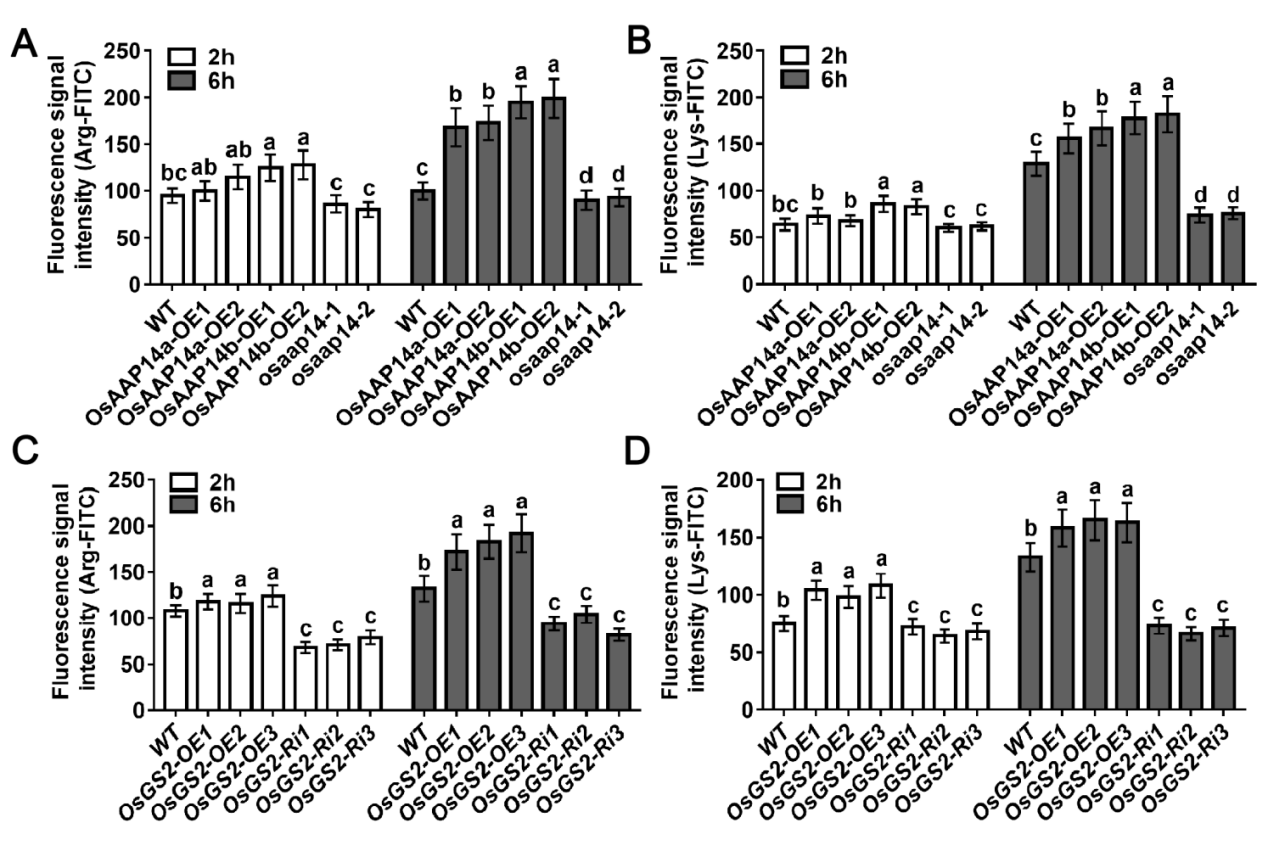


**Supplementary Figure 24.** Statistical analysis of fluorescence signal intensity for Supplementary Figure 20. Fluorescence was detected after culturing seedlings with FITC-labeled amino acids Arg (A) and Lys (B) for 2 h and 6 h of *OsAAP14* transgenic plants, and Arg (C) and Lys (D) for 2 h and 6 h of *OsGS2* transgenic plants. WT represents wild-type ZH11, *OsAAP14a-*OE1-OE3 represent three lines of *OsAAP14* longer splicing variant over-expressing transgenic plants, *OsAAP14b-*OE1-OE3 represent three lines of *OsAAP14* shorter splicing variant over-expressing transgenic plants, *osaap14-1-3* represent three lines of *OsAAP14* CRISPR mutants, *OsGS2* OE1-OE3 represent three lines of *OsGS2* over-expressing transgenic plants, and *OsGS2* Ri1-Ri3 represent three lines of *OsGS2* Ri transgenic plants. Lys represents lysine, and Arg represents arginine. Fluorescence intensities were normalized to the respective area by ImageJ software. Values are means ± SD (n > 6), and the significance levels of different lowercase letters were as follows, *P* < 0.05.
